# Polyunsaturated fatty acids and fatty acid-derived lipid mediators: Recent advances in the understanding of their biosynthesis, structures, and functions

**DOI:** 10.1016/j.plipres.2022.101165

**Published:** 2022-05-01

**Authors:** Simon C. Dyall, Laurence Balas, Nicolas G. Bazan, J. Thomas Brenna, Nan Chiang, Felipe da Costa Souza, Jesmond Dalli, Thierry Durand, Jean-Marie Galano, Pamela J. Lein, Charles N. Serhan, Ameer Y. Taha

**Affiliations:** a School of Life and Health Sciences, University of Roehampton, London, UK; b IBMM, University Montpellier, CNRS, ENSCM, Montpellier, France; c Neuroscience Center of Excellence, School of Medicine, Louisiana State University Health New Orleans, New Orleans, LA, USA; d Depts of Pediatrics, of Chemistry and of Nutrition, Dell Pediatric Research Institute, University of Texas at Austin, TX, USA; e Division of Nutritional Sciences, Cornell University, Ithaca, NY, USA; f Center for Experimental Therapeutics and Reperfusion Injury, Department of Anesthesiology, Perioperative and Pain Medicine, Brigham and Women’s Hospital and Harvard Medical School, Boston, MA, USA; g Department of Molecular Biosciences, School of Veterinary Medicine, University of California-Davis, Davis, CA, USA; h Department of Food Science and Technology, College of Agriculture and Environmental Sciences, University of California, Davis, CA, USA; i William Harvey Research Institute, Barts and The London School of Medicine and Dentistry, Queen Mary University of London, London, UK

**Keywords:** Elovanoids, FAHFA, Fatty acid desaturase, Lipid mediators, Maresins, Omega-3 PUFA, Oxylipins, Protectins, Resolvins, SPM

## Abstract

Polyunsaturated fatty acids (PUFAs) are structural components of membrane phospholipids, and influence cellular function via effects on membrane properties, and also by acting as a precursor pool for lipid mediators. These lipid mediators are formed via activation of pathways involving at least one step of dioxygen-dependent oxidation, and are consequently called oxylipins. Their biosynthesis can be either enzymatically-dependent, utilising the promiscuous cyclooxygenase, lipoxygenase, or cytochrome P450 mixed function oxidase pathways, or nonenzymatic via free radical-catalyzed pathways. The oxylipins include the classical eicosanoids, comprising prostaglandins, thromboxanes, and leukotrienes, and also more recently identified lipid mediators. With the advent of new technologies there is growing interest in identifying these different lipid mediators and characterising their roles in health and disease. This review brings together contributions from some of those at the forefront of research into lipid mediators, who provide brief introductions and summaries of current understanding of the structure and functions of the main classes of nonclassical oxylipins. The topics covered include omega-3 and omega-6 PUFA biosynthesis pathways, focusing on the roles of the different fatty acid desaturase enzymes, oxidized linoleic acid metabolites, omega-3 PUFA-derived specialized pro-resolving mediators, elovanoids, nonenzymatically oxidized PUFAs, and fatty acid esters of hydroxy fatty acids.

## 1. Introduction

Historically, lipids have been associated with two basic functions, as structural components of membranes and a source of metabolic energy. A third function as signalling and regulatory “bioactive lipid” molecules has more recently emerged, where a change in the concentration of the lipid leads to alterations in cellular function. A wide variety of lipids have been shown to exhibit bioactive properties, including glycerolipid-derived molecules, such as phosphatidic acid, monoacylglycerols, lyso-phosphatidic acid, and platelet activating factor; the sphingolipids, such as ceramide, sphingosine, sphingosine-1-phosphate, ceramide-1-phosphate and lyso-sphingomyelin; and the endocannabinoids [1–4]. However, although the bioactive role of lipids has only recently been more widely appreciated, the field of bioactive lipids arguably began in 1935 with the seminal work of Ulf Svante von Euler, who first identified the actions of substances that he would name “prostaglandins” [5]. Following the structural elucidation of prostaglandins in the early 1960s by Bergstrŏm, Samuelsson, and co-workers, the omega-6 polyunsaturated fatty acid (PUFA), arachidonic acid (ARA, 20:4n-6) was identified as the precursor of series 2 prostaglandins, and soon after dihomo-γ-linolenic acid (DGLA, 20:3n-6) and eicosapentaenoic acid (EPA, 20:5n-3) were shown to form series 1 and series 3 prostaglandins, respectively [6]. Since this early work vast repertoires of fatty acid-derived bioactive lipid mediators have been identified.

Fatty acids undergo a wide variety of chemical modifications to greatly expand their functional repertoire and biological activities. The term “oxylipin” was introduced in 1991 to refer to fatty acid-derived oxygenated compounds produced by at least one mono- or dioxygenase oxygenations [7], and is now used to encompasses a very wide variety of bioactive lipid mediators. Oxylipins can be formed either via enzymatic or nonenzymatic free-radical-catalyzed pathways. Three main enzymatic pathways involved in the production of oxylipins are; 1) cyclooxygenase (COX, prostaglandin endoperoxide synthase, prostaglandin H synthase) and subsequent synthases; 2) lipoxygenase (LOX); and 3) cytochrome P450 mixed function oxidase enzymes (CYP) [8], which are described in Section 2. Oxylipins derived from C18 PUFAs, such as linoleic acid (LA, 18:2n-6) and α-linolenic acid (ALA, 18:3n-3) are octadecanoids, whereas those derived from C20 fatty acids such as ARA, DGLA and EPA are eicosanoids, and the classical eicosanoids include the prostaglandins, thromboxanes, and leukotrienes. Docosanoids are derived from C22 fatty acids, such as adrenic acid (AdA, 22:4n-6), docosapentaenoic acids (DPAs, 22:5n-3 or 22:5n-6), and docosahexaenoic acid (DHA, 22:6n-3) [4].

The focus of this review is on the more recently identified enzymatically and nonenzymatically derived oxylipins, and consequently does not discuss the classical eicosanoids. Readers interested in this topic are directed to a number of excellent reviews [9–11]. This review brings together contributions from those at the forefront of their respective fields and reviews current understanding of the structure and functions of the main classes of nonclassical oxylipins, with particular focus on those derived from omega-3 and omega-6 PUFAs. The review begins with an overview of enzyme systems responsible for oxylipin biosynthesis, and then details the biosynthesis of the long-chain omega-6 and omega-3 PUFAs and recent work investigating the role of fatty acid desaturase enzymes in this process, before moving to octadecanoids, particularly those derived from LA, the specialized pro-resolving mediators (SPMs) derived from EPA, DPAn-3 and DHA, elovanoids (ELVs) derived from very long-chain PUFAs, nonenzymatically derived oxylipins, and concludes with the fatty acid esters of hydroxy fatty acids (FAHFAs).

## 2. Enzymatic oxylipin biosynthesis

Enzymatic biosynthesis of oxylipins occurs via multistep processes involving a range of pathways, which are initiated by the initial de-esterification of the fatty acids from membrane phospholipids, catalysed by enzymes from the phospholipase A2 superfamily [12,13]. In the following section a brief overview of the role of the cyclooxygenase (COX), lipoxygenase (LOX) and cytochrome P450 (CYP) enzymes in the biosynthesis of oxylipins is presented. For more detailed coverage the following reviews provide excellent coverage of the topic [10,14–16].

### 2.1. Cyclooxygenase

Cyclooxygenases (COXs) are heme-containing enzymes possessing both oxygenase and peroxidase activities. COX catalyses the initial oxygenation of non-esterified fatty acids to produce prostaglandin H (PGH), a short-lived intermediate, which is further metabolised into prostanoids, such as the prostaglandin D, E and F series (PGD, PGE, PGF), prostacyclins (PGI), thromboxanes, and hydroxy fatty acids [17]. Vertebrates have two principal COX isoforms: COX-1 and COX-2 [18]. COX-1 is constitutively expressed in nearly all tissues, although particularly in blood vessels, smooth muscle cells, interstitial cells, platelets, and mesothelial cells [19]. COX-2 is an inducible enzyme in most tissues in response to inflammatory stimuli; however, constitutive expression has been observed in blood vessels, brain, gastrointestinal tract, kidney, lung, and thymus [20]. COXs oxygenate a wide range of unsaturated fatty acids and fatty esters [21].

### 2.2. Lipoxygenase

Lipoxygenases (LOXs) are a family of dioxygenases that catalyse the formation of hydroperoxyl fatty acids and their metabolites, such as leukotrienes, lipoxins, and the SPMs, including resolvins (Rvs), protectins (PDs) and maresins (MaRs) derived from various omega-3 PUFAs [4], which are described in detail in Section 4.

There are six functional LOX genes in the human genome, which are expressed across a range of tissues [15]. LOXs were traditionally classified based on the position of the hydroxyl and hydroperoxy fatty acids they produce from ARA e.g., 5-LOX forms 5-hydroperoxy-eicosatetraenoic acid (5-HpETE), but not 5-hydroxyeicosatetraenoic acid (5-HETE), the latter being obtained through reduction of 5-HpETE by glutathione peroxidase. However, this nomenclature has limitations as the position varies according to different chain lengths of the substrates and some LOXs act at more than one position [11]. Furthermore, the most recently characterised LOX, epidermis-type lipoxygenase 3 (eLOX3) is unconventional in that it has limited lipoxygenase activity, and therefore the addition of gene names in addition to the enzyme name has been suggested [15]. Lipoxins, Rvs, PDs, and MaRs are formed by combinations of LOX activities and sequential epoxygenase and hydrolase activities, which generate epoxyalcohols (hepoxilins) and epoxyketones (eoxins). Hepoxylins are formed from 12-HpETE and eoxins from 15-HpETE and hepoxilins are epoxyalcohols, and eoxins are 14,15-analogs of leukotrienes [4].

A further class of metabolites generated from omega-3 PUFAs by LOX are the electrophilic fatty acid oxo-derivatives (EFOX), with 7-oxo-DHA,7-oxo-DPAn-3 and 5-oxo-EPA produced from DHA, DPAn-3 and EPA, respectively [22,23]. EFOXs display a wide range of anti-inflammatory actions, including acting as agonists nuclear receptors, such as the peroxisome proliferator-activated receptors (PPAR) and inhibiting cytokine production in activated macrophages [23].

### 2.3. Cytochrome P450 mixed function oxidase

The third oxidative PUFA pathway involves the cytochrome P450 mixed function oxidase (CYP) enzyme activity as monooxygenases and catalysing epoxidation, hydroxylation or allylic oxidation reactions, which metabolise PUFAs to lipid mediators with many diverse biological functions at both the systemic and cellular levels [24,25]. Regio- and stereoisomers of epoxyeicosatrienoic acids (EETs) and hydroxyeicosatetraenoic acids (HETEs) are produced from ARA, whereas those derived from EPA include epoxyeicosatetraenoic acids (EpETEs) and hydroxyeicosapentaenoic acids (HEPEs), and epoxydocosapentaenoic acids (EDPs) and hydroxydocosahexaenoic acids (HDoHEs) from DHA [25]. EPA is the preferred substrate for the majority of CYP isoforms, with DHA and ARA metabolized at similar rates [25].

## 3. Omega-3 and Omega-6 PUFA Biosynthesis

**Fig. 1.**
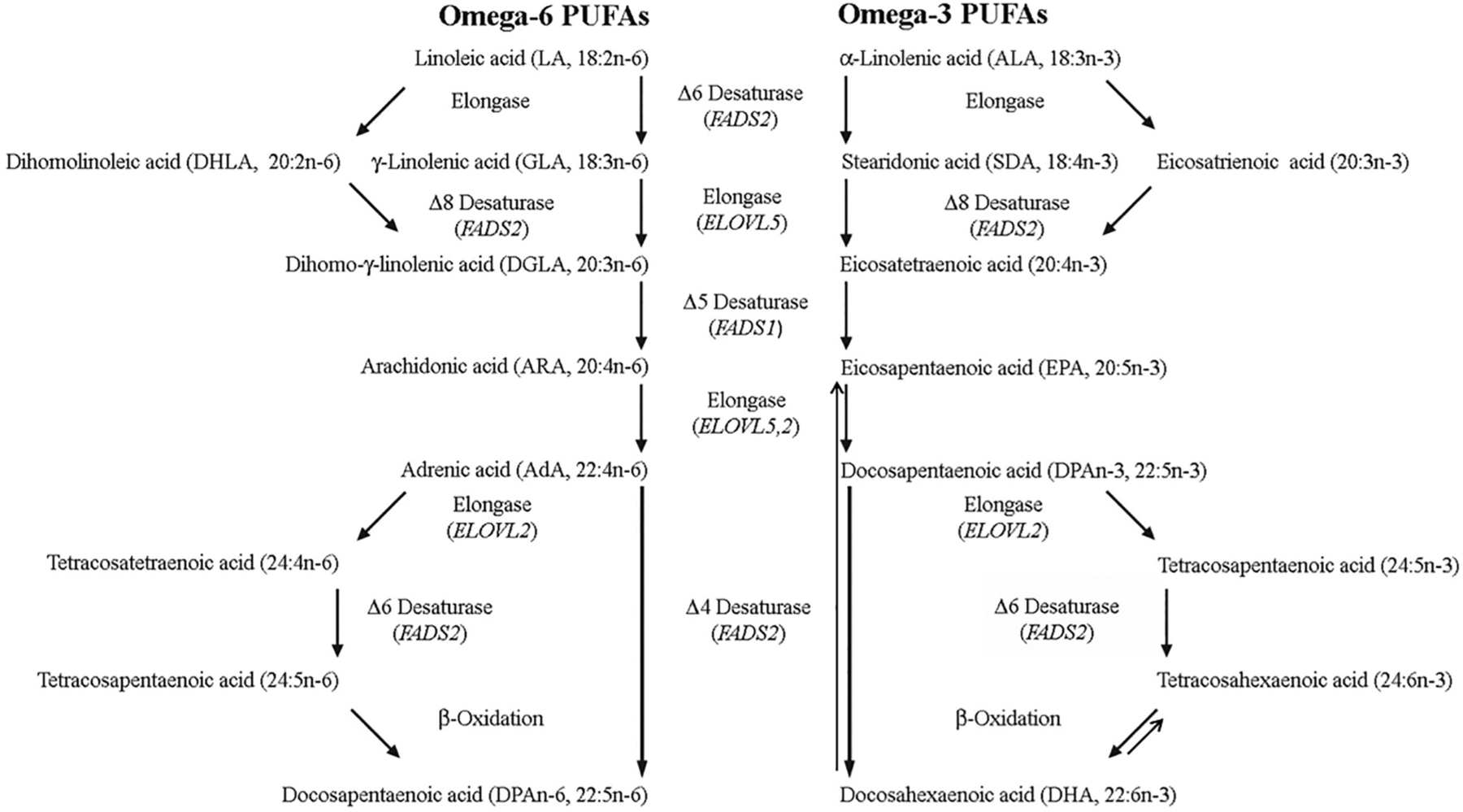
Biosynthesis of omega-3 and omega-6 PUFAs. The biosynthesis of longer-chain omega-3 and omega-6 PUFAs proceeds via a series of alternating position-specific desaturation and elongation steps from ALA and LA, respectively. FADS1 and FADS2 appear responsible for all omega-3 and omega-6 PUFA desaturation in mammals, with FADS1 exhibiting Δ5-desaturase activity, and although FADS2 was originally identified as the Δ6-desaturase, it has subsequently been shown to also possess Δ4- and Δ8-desaturase activities. Octadecanoids are lipid mediators derived from C18 PUFAs, such as ALA or LA, eicosanoids are derived from C20 PUFAs such as DGLA, ARA or EPA, and docosanoids are derived from C22 PUFAs such as DPAn-3, and DHA. See text for further details.

Since lipid mediators originate from PUFAs, this section will cover their *in vivo* synthetic pathways. The omega-3 and omega-6 families of PUFAs were first named by Ralph T. Holman [26], and the biosynthesis of their longer-chain versions, such as ARA and DHA, proceeds via a series of alternating position-specific desaturation (fatty acid desaturase genes, FADS1 and FADS2, located at 11q12–13.1) and elongation (elongase genes, ELOVL2 at 6p24.2 and ELOVL5 at 6p12.1) steps from LA and ALA, respectively, and are summarised in Fig. 1. *In the following section J.T. Brenna describes the biosynthesis of PUFAs and the role of fatty acid desaturase enzymes (FADS)*.

Fatty acids were likely the first metabolites that were routinely reported as a panel of metabolites because of the early development of gas chromatography [27]. PUFA desaturation was presumed to be mediated by three desaturation enzymes known as the 6, 5, and 4 desaturases. However, because of the wide variety of similar structural fatty acids, the specificity of these enzyme activities awaited the widespread availability of molecular tools. In recent years we have investigated the structural specificity of the FADS genes using specialized tools.We used genetically transformed yeast or human cells, both devoid of 6-desaturase activity, and a facile method for unambiguously determining double bond position without chemical standards [28,29] to investigate FADS1, FADS2, and FADS3 structural specificity. In our transformed human cell experiments, human MCF-7 cells were transformed to stably express FADS1, FADS2 or an empty vector to investigate the function of FADS1 and FADS2 classical transcripts. In nearly all studies, all three cell lines were incubated with a single test fatty acid and the product measured. In this way we could be confident that findings of no activity in one enzyme vs. activity in the other was reflective of differential specificity. We have also discovered functions of alternatively spliced (nonclassical transcripts), beyond the scope of this brief review [30].

FADS3 is not a PUFA desaturase.The fatty desaturase gene cluster on chromosome 11 arose by gene duplication, and consists of three similar genes:FADS1, FADS2, and FADS3 [31].Each consists of 12 exons and 11 introns, with FADS1 and FADS2 arranged head-to-head upstream of FADS3.FADS 1 and FADS2 were identified as catalyzing Δ5-desaturation [32] and Δ6-desaturation [33], respectively, in early work.Because of its genetic similarity, FADS3 was assumed to have PUFA desaturase activity.However, extensive searches for its substrates led to only two functions, one against a relatively rare fatty acid, and the other a global effect.FADS3 was identified as a “back-end” desaturase that catalyzed the conversion of vaccenic acid (11E-18:1) to the conjugated 11E,13Z-18:2 in the rat mammary gland [34].Vaccenic acid is the most abundant *trans* fatty acid in cow’s milk, though the diene product is below 0.1% of fatty acids in rat milk.Alterations in the fatty acid profiles of brain tissue in FADS3 knockout mice was also reported [35].Recently, with the aid of FADS3-knockout mice, FADS3 was shown to be a Δ14-desaturase for the sphingoid base, yielding 4E,14Z-sphingodienine [36], apparently consistent with its role as a back-end desaturase.This precursor/product pair is readily detected in normal tissue, thus showing FADS3 *in vivo* is not a desaturase for PUFAs. Thus, FADS1 and FADS2 appear to be responsible for all PUFA desaturation in mammals. Compared to their classical biochemical roles, the specificities of the classical transcripts are FADS1 and FADS2 are very different in their range of specificities and substrates, with FADS1 far more specific than FADS2.

In mammalian systems, FADS1 and FADS2 activity toward a particular omega-6 PUFA always shows activity toward the n-3 structural analogue, and usually at a higher kinetic rate.That is, presence of a double bond at the n-3 position increases activity.As a shorthand we will refer to substrates as pairs.

### FADS1.

FADS1 Δ5-desaturase activity appears to be entirely toward C20 PUFAs.The major activity is toward 20:3n-6/20:4n-3 to yield 20:4n-6 (ARA) and 20:5n-3 (EPA), respectively; in essential fatty acid deficiency, FADS1 Δ5-desaturates 20:2n-9 to 20:3n-9 (Mead acid).When 6 desaturase activity is absent, FADS1 Δ5-desaturates 20:2n-6 to 5,11,14–20:3 (sciadonic acid), a structural analogue of ARA (5,8,11,14–20:4).Owing to the absence of the double bond at position 8–9, sciadonic acid is not a substrate for synthesis of prostaglandins, leukotrienes, or thromboxanes. Sciadonic acid is found in MCF-7 human breast cancer cells [37], long known to have no Δ6-desaturase activity, as well as in vivo in human breast cancer biopsies [38].Cats naturally have very low Δ6-desaturase activity [39] and make 5,11,14–20:3, and possibly 7,11,14–20:3 via elongation of a Δ5-desaturated 11,14–18:2, as originally reported [40].

We reported evidence that FADS1 can insert a double bond at position 7 to make a rare PUFA, 7,11–20:2 [41].FADS1 has no activity toward normal or branched chain (iso, anteiso) saturated fatty acids [42]. Integrating the evidence for FADS1 function and genetics, FADS1 appears to function primarily to synthesize and regulate the levels of its immediate product, the key eicosanoid precursor fatty acid, ARA. In this respect, FADS1 might be accurately called “ARA synthase”.

### FADS2.

FADS2 was originally identified as the Δ6-desaturase catalyzing 18:2n-6/18:3n-3 conversion to 18:3n-6/18:4n-3.FADS2 was first identified as having Δ8-desaturase activity toward 20:2n-6/20:3n-3 to make 20:3n-6/20:4n-3 [43].Later, FADS2 was shown to have Δ4-desaturase activity toward 22:4n-6/22:5n-3 to yield 22:5n-6/22:6n-3 [44]. This activity is shown most clearly for 22:4n-6/22:5n-6 in pulse-chase experiments because of the low level of endogenous substrates.Synthesis of DHA via this pathway was shown by similar pulse-chase methods as well as isotope labeling, consistent with Δ4-desatuase activity in many organisms [45].FADS2 was recently shown in an unambiguous manner to desaturate 24:4n-6/24:5n-3 to 24:5n-5/24:6n-3 [46]; this step is required in the Sprecher pathway of 22:5n-6/22:6n-3 synthesis.

FADS2 has activity toward the saturated fatty acid 16:0 to make 16:1n-10 (sapienic acid), the most abundant unsaturated fatty acid on human skin, but seldom reported in internal tissue.FADS2 has no detectable activity toward 14:0 or 18:0. Based on competition experiments, we predicted that high levels of saturated fatty acids due to metabolic derangement would result in production of 16:1n-10 [47], recently confirmed in carcinoma [48,49].We recently followed up those experiments with saturated odd and branched chain fatty acids.Those studies show that FADS2 is active toward n-17:0, iso-16:0, iso-17:0, anteiso-17:0, iso-18:0 [42], inserting double bonds between carbons 6 and 7 and yielding the series of monounsaturated odd and branched chain fatty acids in human sebum.All told, FADS2 is active toward at least 16 substrates and inserts double bonds in the 4, 6, and 8 positions, far more promiscuous than FADS1.

## 4. Bioactive lipids mediators derived from PUFAs

### 4.1. Octadecanoids

There has been a large and rapid increase in the amount of LA consumed Worldwide, due to the agricultural shifts towards high-LA soybean and corn oils since the late 1930s [50]. Historic levels of LA intake ranged between 1 and 2% of daily calories pre-1930s, to the current situation where the average is greater than 7%, and LA is now the most highly consumed PUFA in Western diet [50]. LA has been shown to be the precursor to oxylipins, called oxidized LA metabolites, (OXLAMs), which have been linked to a range of pathological conditions [51]. Consequently, LA-derived octadecanoids are quantitatively the major class of oxylipins present in tissues and blood; however, compared to some of the other classes there is much less known about their functions [11], although early investigations indicated that in familiar Mediterranean fever, C18 oxygenated compounds were identified and elevated [52]. *In the following section F. da Costa Souza, P. Lein, and A. Taha provide an overview of the structure and functions of oct*a*decanoids, with particular focus on the LA-derived OXLAMs*.

In 1929, George Burr and Mildred Burr settled a long-standing debate at the time on the essentiality of dietary fats [53]. They conclusively established that LA, along with ALA, are nutritionally essential [54]. In humans, LA is needed at 1–2% daily energy to maintain optimal growth and skin barrier integrity [55], whereas ALA is needed at 0.2–0.5% energy, also to maintain the skin barrier as well as brain function [56,57]. It is becoming increasingly recognized that the biological effects of LA and ALA are mediated through their oxidized lipid mediator products. This section will focus on LA because it is highly abundant in the diet and because its lipid-mediated roles *in vivo* remain understudied.

For many decades, LA’s presumed biological role was as a substrate for the synthesis of ARA via elongase and desaturase enzymes. ARA itself is not nutritionally essential but serves key biological roles *in vivo* through its enzymatically-derived oxidized metabolites, including prostaglandin E2, an immune modulator [58], and prostaglandin F2-alpha, which regulates blood flow [59].

Research in the 1980s provided evidence that LA is not only a precursor to ARA, but also to bioactive lipid autacoids known as OXLAMs. OXLAMs can be formed via auto-oxidation or COX [60,61], LOX [62,63], CYP [64], 15-Hydroxyprostaglandin Dehydrogenase [65] and soluble epoxide hydrolase (sEH) enzymes [66–68]. Notably, these are the same enzymes used to oxidize ALA and other PUFAs into bioactive lipid mediators. LOX and COX catalyze the addition of a hydroxy group to LA or ALA, while 15-PGDH converts hydroxylated LA or ALA into fatty acid ketones. CYPs produce epoxidized fatty acids that can be converted into diols by sEH. Examples of OXLAMs include LOX-derived 9- and 13-hydroxyoctadecadienoic acids (HODEs), their ketone metabolites, 9- and 13-oxo-octadecadienoic acids, as well CYP-derived epoxyoctadecamonoenoic acids (EpOMEs), and sEH-derived dihydroxyoctadecamonoenoic acids (DiHOMEs). Similarly, ALA oxidation through the same enzymatic machinery yields hydroxyoctadecatrienoic acids (HOTrEs), epoxyoctadecadienoic acid (EpODEs) and dihydroxyoctadecadienoic acid (DiHODEs). Some ALA-derived oxylipins may have anti-inflammatory effects *in vitro* (e.g., 13-HOTrE) [69], yet the role and tissue distribution of several ALA metabolites remains unknown due to the lack of analytical standards.

The long-standing question in the field is whether OXLAMs are bioactive. Early studies in the 1950s showed that chicks fed a diet containing LA without added vitamin E developed encephalomalacia and ataxia [70–72]. It was realized that the absence of vitamin E in the diet promoted the nonenzymatic oxidation of LA into OXLAMs. Adding purified OXLAMs to the diet induced similar pathological and behavioural symptoms in chicks, suggesting a direct influence of OXLAMs on brain function [73,74]. Additional studies by Hammock and colleagues showed that sEH-derived linoleate diols (known as leukotoxin diols) are cytotoxic and promote inflammation in rats and mice [75,76]. Other studies showed that OXLAMs are involved in lowering pain thresholds by binding TRPV-1 receptors [77], and in maintaining skin barrier integrity [78]. An early study also identified 13-HODE, a LOX-derived OXLAM, functioned as a chemorepellent to platelet adhesion in endothelial cells [79]. Collectively, the evidence suggests that OXLAMs are bioactive *in vivo* and *in vitro*.

Our group has been interested in knowing whether OXLAMs are present and bioactive in the brain, where they have been rarely studied. Despite being a major part of the diet [50], LA is not found in appreciable levels in the brain (<2% of total fatty acids) because it is mostly (~60%) beta-oxidized upon entry [80]. Thus, we questioned whether a portion of the LA that enters the brain is converted into OXLAMs, having established that OXLAMs are unlikely to cross the blood brain barrier in adult rats [81].

Our experiments showed that brain LA serves as a precursor to OXLAMs, but the extent of conversion depends on life stage. In adult rats, OXLAMs constitute 7% of detected oxylipins in the brain, and this value increases as dietary LA increases, suggesting that brain OXLAM concentrations are dependent on dietary LA levels [82]. Hence, more LA in the diet means more LA entering the brain and more OXLAMs synthesized there. In young rat pups (0–1 day old males and females), we unexpectedly found that OXLAMs constituted 50% of brain oxylipins. We do not yet know whether the higher brain OXLAM composition observed in younger rats originates from diet (which contains OXLAMs) or conversion of dietary LA entering the brain into OXLAMs.

Our studies led us to further investigate the role of OXLAMs in the adult and developing rat brain. We found that in adults, OXLAMs are produced during global ischemia, similar to AA-derived prostanoids (and other eicosanoids), raising the possibility that they might be involved in the response to ischemic brain injury [83,84]. They also increase somatic pre-pulse facilitation, suggesting their involvement in neurotransmission [84]. In young pups, OXLAMs such as 13-HODE were shown to increase axonal outgrowth in primary rat cortical neurons derived from 0 to 1 day old male pups, providing evidence of their involvement in neuronal morphogenesis in early life [85].

Overall, the evidence to date shows that OXLAMs are bioactive lipid mediators involved in regulating pain thresholds, inflammation, response to ischemic brain injury, neurotransmission and neuronal morphogenesis. However, many scientific gaps remain with respect to their newly identified roles in the brain. Specifically, what are the mechanisms mediating their effects in the brain? If brain OXLAM composition is dependent on dietary LA levels (at least in adult rats), what does this mean in terms of benefit or harm to the brain, given that dietary LA levels have increased by more than three-fold over the past century? At what stage of life (e.g., development versus aging) are these OXLAMs beneficial (or harmful) to the brain? Answering these questions will help calibrate dietary LA to levels that optimize brain function during development, adulthood and aging. The Burrs discovered that LA is essential at 1–2% energy; the question is what may be optimal beyond this level of dietary intake?

### 4.2. n-3 PUFA derived specialized pro-resolving mediators (SPMs) and Receptors

In the following sections N. Chiang and C.N. Serhan provide a brief overview of the current status of EPA- and DHA-derived SPMs and their biological functions in inflammation-resolution. This followed by J. Dalli providing an overview of SPMs derived from DPAn-3. For ARA-derived lipoxins biosynthesis and functions, please see earlier in-depth reviews [86–89].

#### 4.2.1. Resolution phase mediators in inflammation

Resolution of inflammation is an active biosynthetic process that connects the first response of the innate immune system to biosynthesis of the SPMs including Rvs, PDs and MaRs, as well as novel cys-SPMs [90]. The first resolvin biosynthesized from EPA was isolated and reported along with functional elucidation in 2000 using a systems approach with inflammatory exudates [91]. As of today, four potent bioactive resolvins produced from EPA (E-series resolvins) have been elucidated [92]. The DHA-derived resolvins were elucidated next and reported in [93,94]. The D-series resolvins and protectins biosynthesized from DHA were initially demonstrated to reduce inflammation (peritonitis), neuronal inflammation (microglial cells), and counter-regulate cytokines and chemokines to promote resolution of inflammation. Next, we systematically determined the stereochemical assignments of each of the potent bioactive SPMs, including protectins [95], and their aspirin-triggered epimers [96], and next reported the discovery and biosynthesis of the maresins from infiltrating macrophages [97]. Today in PubMed.gov with “resolvin” as the search term there are more than 1400 publications reporting the potent anti-inflammatory and pro-resolving actions as well as productions of the resolvins, protectins, and maresins in many disease models from investigators worldwide that confirm and extend the potent endogenous functions of SPMs and their potential in novel therapeutics as agonists of resolution of inflammation originally described for each SPM [98–100]. An early consensus report helped to define and underscored the potential of this new field of resolution with impact in modern medicine and surgery [101]. The emergence of new concepts and novel mediators within the resolution terrain that activate endogenous resolution programs and promote tissue regeneration have given rise to the new fields of resolution pharmacology and physiology. For readers interested in further details in in-depth reviews, please see [102–107].

#### 4.2.2. SPMs in human tissues and dysregulation in diseases

Mass spectrometry-based profiling approaches for the resolution metabolome have documented the temporal production of SPMs in humans (Table 1A) and preclinical animal systems, demonstrating *in vivo* the lipid mediator class switch. For example, human vagus nerves produce SPMs, e.g., RvE1, NPD1/PD1, MaR1, upon electrical stimulation [108] suggesting that this vagus-SPM circuits contribute to a new proresolving vagal reflex. Several clinical trials demonstrate omega-3 PUFA or marine oil supplementation increase SPM *in vivo* [109] (Table 1B). SPM biosynthesis is impaired in several diseases, including tuberculous meningitis [110], multiple sclerosis [111], and osteoarthritis [112], as well as in bronchoalveolar lavages [113], serum [114] and plasma [115] from COVID-19 patients. Thus, impaired endogenous resolution pathways may contribute to the pathogenesis of these diseases.

#### 4.2.3. SPM functions and receptors

**Fig. 2.**
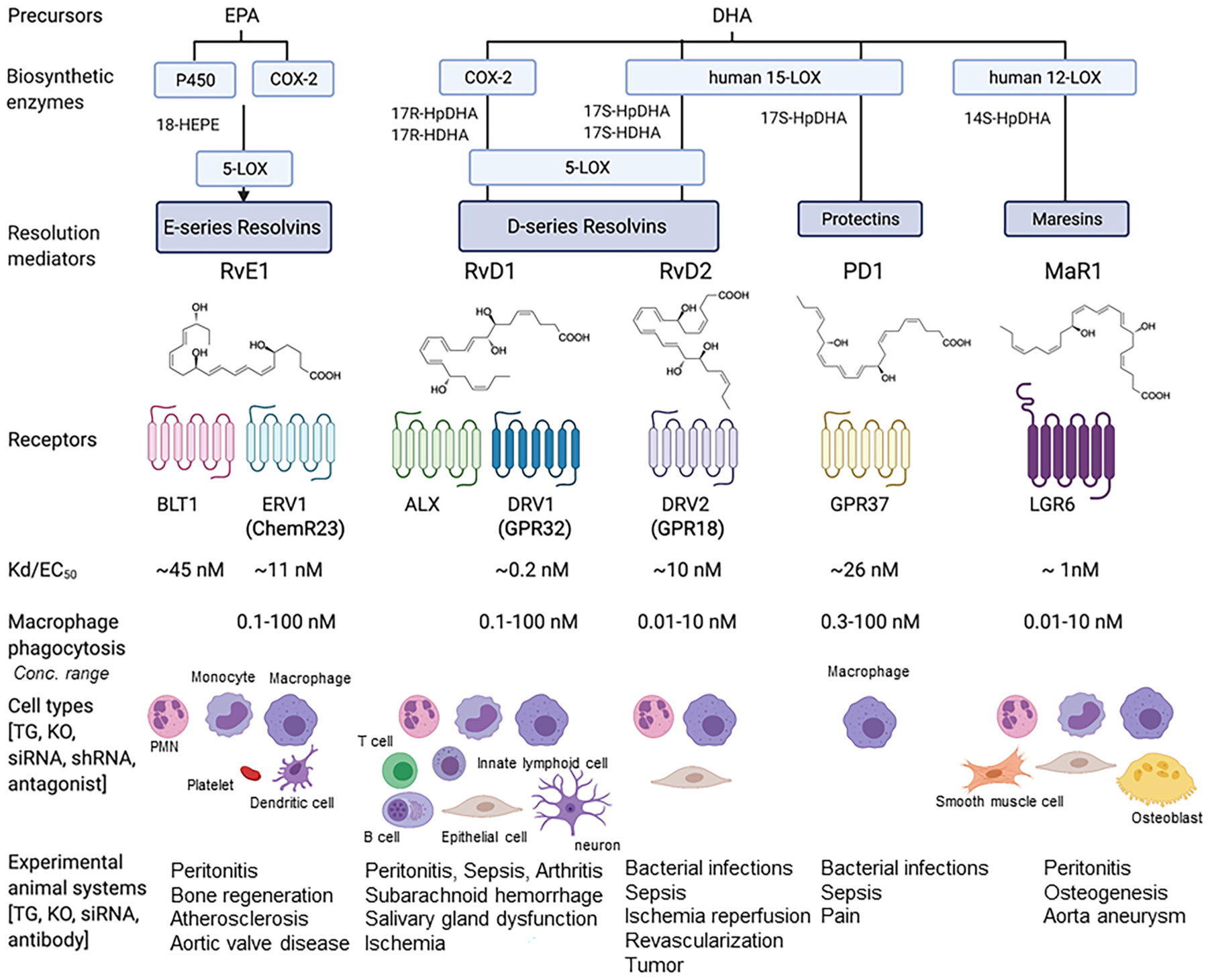
Illustration of resolution metabolome: SPM biosynthesis, receptors and functions. Precursors EPA and DHA are converted via biosynthetic enzymes to SPMs, which in turn activate their specific receptors to stimulate pro-resolving innate immune functions. Each SPM demonstrates stereoselective activation of its cognate GPCR on select cell types, leading to intracellular signals, pathways and pro-resolving functions The affinities of SPMs for their respective recombinant GPCRs (i.e., Kd or EC 50 values) are consistent with their bioactive concentration ranges, e.g. macrophage phagocytosis (picomolar to low nanomolar) *in vitro* and dose ranges (picograms to low nanograms) *in vivo*.The *in vivo* functions of these SPM receptors were demonstrated using transgenic and/or knock-out mice, as well as specific blockage of the receptor, e.g., siRNA, antibodies or receptor antagonists (see text and recent reviews [98,116] for details).

Each SPM demonstrates potent stereoselective actions (pico- to low nanomolar concentrations) via activation of specific G protein-coupled receptors (GPCR) on phagocytes and additional select cell types (Fig. 2).

##### Resolvins:

Resolution phase interaction products.

##### E-series resolvins:

RvE1 was the first identified pro-resolving molecule derived from EPA [91]. RvE1 via its receptor ERV1/ChemR23 (Kd ~11 nM) stimulates intracellular signals such as phosphorylation of S6 kinase (0.1–100 nM) (Fig. 2; reviewed in [116]). RvD1, *in vivo*, controls vascular inflammation, protecting against atherosclerosis by modifying oxidized LDL uptake and enhancing macrophage phagocytosis [117]. In aortic valve stenosis, targeted deletion of ChemR23 in mice heightens disease progression [118]. Of interest, an agonist antibody to the RvE1 receptor confirms that activation of the endogenous resolution mechanisms can control both inflammation and cancer burdens in mouse models *in vivo* [119].

##### D-series resolvins:

RvDs are biosynthesized from DHA; they are potent immunoresolvents active in the picomolar to low nanomolar concentrations [93,94]. RvD1 binds and activates human DRV1/GPR32 (Kd ~0.2 nM) to stimulate macrophage phagocytosis and efferocytosis (0.1–100 nM) (Fig. 2; reviewed in [101]). Some of the most exciting and unexpected findings at the time were the novel actions of RvD2. RvD2 (0.01–10 ng/mouse) limits PMN infiltration in acute inflammation and controls bacterial sepsis via its receptor DRV2/GPR18 in mice (Fig. 2, and review [98]). RvD2 binds and activates human recombinant receptor DRV2/GPR18 (Kd ~10 nM) to stimulate macrophage phagocytosis and efferocytosis (0.01–10 nM). In human sepsis, survivors had a higher percentage of GPR18-positive peripheral blood neutrophils compared to non-survivors, suggesting that DRV2/GPR18 expression levels are associated with disease severity [120]. In a more recent study, both DRV1 and DRV2 receptor expression were found to be higher on leukocytes from septic patients; both RvD1 and RvD2 partially reverse sepsis-induced leukocyte activation, and stimulate phagolysosome formation [121]. RvD2 suppresses tumor growth and enhances clearance of tumor cell debris, while DRV2/GPR18-deficient mice display defective tumor clearance [122]. In addition, RvDs are tissue/organ protective; RvD2 promotes keratinocyte repair in DRV2-dependent manner [123] and stimulates muscle regeneration [124], as well as limits tissue necrosis in burn wound [125]. RvD4 reduces thrombus burden and decreases the release of neutrophil extracellular traps (NETs), i.e. NETosis, a critical component for thrombosis development [126]. These new roles of selective RvDs suggest that SPMs could provide an effective strategy in controlling thrombo-inflammatory disease. RvD5 and RvD1controls *E. coli* and *S aureus* infection, by controlling phagocytosis and bacterial killing as well as inflammation arising from collateral tissue damage; together these lower the antibiotic requirements for bacterial clearance [127]. Of interest, RvD5 is the first SPM that shows sex dimorphism in pain regulation, inhibiting pain in male, but not female mice [128].

##### Protectins:

**Fig. 3.**
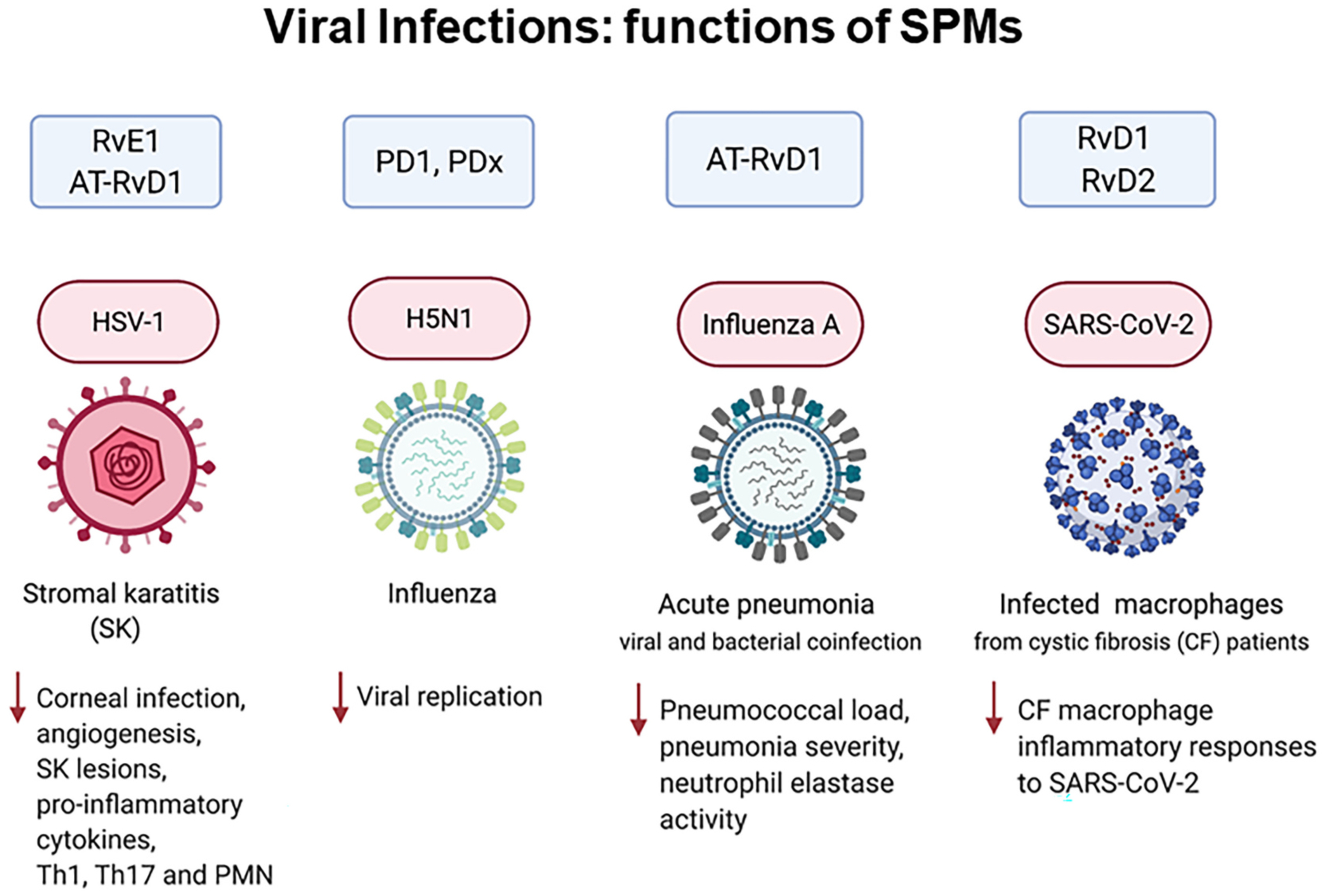
SPM actions in viral infections. See text for details.

Protectin D1/Neuroprotectin D1 (PD1/NPD1) is also biosynthesized from DHA via 15-LOX-initiated mechanism in several human cell types, murine exudates, and brain tissues [94]. In addition, PD1 is present in human exhaled breath condensates, and its levels are lower in subjects with asthma exacerbations [129]. DHA is converted via 15-LOX to the 16S, 17S-epoxide intermediate, confirmed by epoxide trapping experiments. This epoxide intermediate is further converted to PD1 via enzymatic hydrolysis [95]. The elicited bioactivity of this mediator in human retinal pigment epithelial cells led to coining its name as Neuroprotectin D1 (NPD1) [130]. This was strongly supported by the demonstration of its formation in the human brain and its selective decrease in memory areas of the brains of Alzheimer’s patients and in experimental Alzheimer’s disease models [131,132], as well as in experimental ischemic stroke [133]. The complete stereochemical assignments [95] enabled the demonstration of its potent actions on human PMN [1–100 nM] and acute inflammation in vivo [0.01–100 ng/mouse] as well as in many disease systems, confirmed and extended by many other investigators worldwide. Hence, while produced and functions in neural systems, the prefix (neuro)protectin D1 was introduced [130], and in the immune system, it is PD1 [134]. PD1/NPD1 displays potent neuroprotective actions in brain, retina and central nervous system, e.g. protecting from ischemic stroke, retina degenerative disease (for a recent review, see [99]) and traumatic brain injury [135]. NPD1/PD1 activates recombinant and macrophage GPR37 [EC_50_ ~ 26 nM]. Mice lacking this NPD1/PD1 receptor display defects in macrophage phagocytic activity with delayed resolution of inflammatory pain [136]. PD1’s protective actions in multiple models of infections and sepsis are diminished in these *Gpr37* receptor KO mice [137]. PDX is a positional isomer of PD1, biosynthesized via two sequential lipoxygenations [95]. PDx [0.1–10 μM] inhibits platelet activation [138], improves insulin sensitivity [139] and atherosclerosis [140] in type-2 diabetes. Both PDx and PD1 at equal amount suppress replication of influenza virus [141,142] (Fig. 3).A receptor for PDx remains to be identified. It is likely that PD1 and PDx have some overlapping yet distinct actions on select target cells.

##### Maresins:

The macrophage mediators in resolving inflammation. MaR1 was first identified in self-resolving inflammatory exudates and with human macrophages (MΦ) [97] via 12-LOX-initiated mechanisms [143]. The complete stereochemistry of MaR1 was established, its total organic synthesis was achieved and confirmed by several independent teams (reviewed in [99]). MaR1 is pro-regenerative, pro-repair and neuroprotective in a wide range of tissues and organs across phyla (reviewed in [116]). MaR1 activates LGR6 (leucine-rich repeat-containing G protein–coupled receptor 6), a cell surface G protein-coupled receptor [EC_50_ ~ 1 nM] and stimulates key proresolving functions of phagocytes in a LGR6-dependent manner [0.01–10 nM] [144]. In addition, MaR1 inhibit smooth muscle cell activation and attenuate murine abdominal aortic aneurysm via LGR6 signaling [145]. Further, LGR6 is necessary for normal osteogenesis, demonstrated using LGR6-deficient mice, and MaR1 activates LGR6 signaling in osteoblasts [146]. With liver macrophages, MaR1 can also activate ROR-α (retinoic acid-related orphan receptor α), a nuclear receptor that might be relevant in liver pathology [147]. These findings highlight the cell-type specific and receptor-dependent actions of MaR1.

##### Cys-SPMs:

Three series of peptide-lipid conjugated SPMs, i.e., maresin conjugates in tissue regeneration (MCTRs), protectin conjugates in tissue regeneration (PCTRs) and resolvin conjugates in tissue regeneration (RCTRs), are collectively coined cysteinyl-SPMs (cys-SPMs) ([148]; reviewed in [149]). Each series contains three bioactive members that display potent pro-regenerative and pro-repair actions, including stimulating regeneration of freshwater planaria and promoting tissue repair in acute lung injury [148,150]. Using RNA-sequencing of regenerating planaria, we identified cys-SPM-regulated pathways in planaria regeneration, including NF-κB pathways, and an ortholog of human TRAF3. In human macrophages and mouse infections, cys-SPM regulate the TRAF3/IL-10 axis in enhancing phagocyte functions in resolution [151]. In addition, PCTR1 uniquely enhances human keratinocyte migration, and promotes bacterial clearance in mouse skin wound [152]. These model systems give clear evidence for potent actions and structure-function relationships of Cys-SPMs. *In vivo* human results for Cys-SPM actions remain to be identified. Thus, the organ-protective actions of cys-SPMs are evolutionarily conserved across phyla, from primordial lower-phylum species such as planaria to mice and humans.

###### SPMs control infectious inflammation and the innate immune system.

SPMs exhibit potent host-protective actions in bacterial, parasitic and viral infections [90,98,153] (Fig. 3). For example, RvE1 controls herpes simplex virus (HSV)-induced murine ocular inflammation [154]. PD1 and PDx suppress influenza virus replication [141,142]. In bacterial and viral coinfection pneumonia in mice, the aspirin-triggered 17R-epimer of RvD1 (AT-RvD1) enhances clearance of pneumococci in the lungs [155]. With human macrophage from cystic fibrosis patients, RvD1 and RvD2 (10 nM) reduce SARS-CoV-2 induced inflammatory response [156]. In light of COVID-19 pathologies with hyperinflammation of the respiratory and cardiovascular systems as well as coagulopathies [157–159], the anti-inflammatory, pro-resolving, microbial clearing, anti-thrombotic and organ-protective actions of SPMs may be useful in controlling disease severity in SARS-CoV-2 infections and perhaps long-term COVID-19 symptoms (reviewed in [98]).

In summation, the structural elucidation, complete stereochemical determinations and identification of specific receptors for each SPM enable confirmation of their potent actions in controlling inflammatory response, promoting resolution and tissue repair. Endogenous SPMs present in human tissues are within both their bioactive concentration ranges and affinities for cognate receptors (c.f. Table 1 and Fig. 3). Results from these studies opened an opportunity path for interrogating SPM in resolution physiology and pharmacology.

### 4.3. DPAn-3 is precursor to novel bioactive mediators

**Fig. 4.**
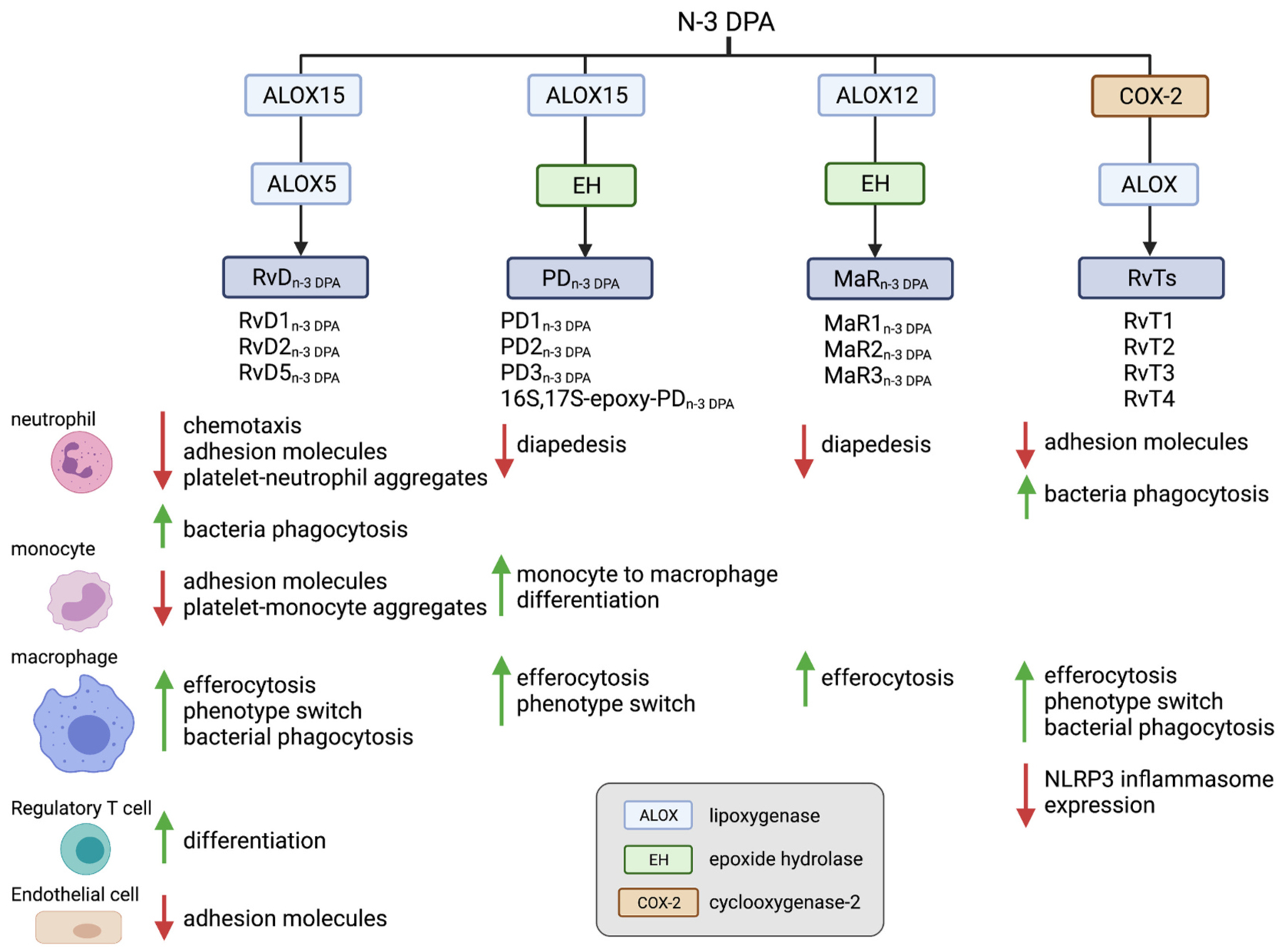
Illustration of the omega-3 DPA derived SPM families and the biological activities exerted on immune and stromal cells. For details of the stereochemistry of the structures of these SPMs please see [162].

A less well studied omega-3 PUFA that forms part of many organs and tissues is DPAn-3 [160]. Genome wide association studies uncovered links between single nucleotide polymorphisms in the gene encoding for the fatty acid elongase 2 (ELOVL2) and increased plasma DPAn-3 levels [161]. Several studies suggested a role for this essential fatty acid in the regulation of inflammation [160]. We recently queried whether the ability of this fatty acid to regulate inflammation was at least in part due to its conversion to novel bioactive mediators. Structure elucidation studies demonstrated that in inflammatory exudates and in the circulation DPAn-3 is converted to bioactive mediators [162]. These novel autacoids exert potent leukocyte directed activities to limit tissue damage by governing cellular recruitment to the site of inflammation and counter regulating the production of pro-inflammatory mediators (Fig. 4). Given that the biosynthetic pathways leading to the formation of these novel molecules are shared with the DHA and EPA derived SPM, these novel bioactive mediators were assigned to the Rv, PD and MaR families [162,163].

#### 4.3.1. DPAn-3-derived SPM in the regulation of acute inflammation

Endogenous formation of these bioactive mediators is reported both during homeostasis and active inflammation. Lipid mediator profiling of plasma lipid mediator concentrations in healthy volunteers uncovered a diurnal regulation in the production of DPAn-3-derived D-series resolvins (RvD_n-3 DPA_) [164]. The peak in the production of these mediators is coincident with an increase in plasma plasminogen activator inhibitor-1, a serine protease inhibitor that functions as the principal inhibitor of tissue plasminogen activator and urokinase, and the activation of circulating platelets and phagocytes thereby increasing the risk of thrombosis [165]. Intriguingly we observed that RvD_n-3_ DPA, when added to blood from healthy volunteer’s *ex vivo*, downregulated the expression of adhesion molecules on circulating phagocytes and the formation of phagocyte-platelet heterotypic aggregates. This vasculo-protective role of RvD_n-3_ DPA was supported by observations made in patients with cardiovascular disease, where both the diurnal regulation and the production of these mediators are reduced [164]. These changes were linked with increased peripheral blood phagocyte and platelet activation. *Ex vivo* incubation of these mediators with peripheral blood from patients with cardiovascular disease downregulated both platelet and phagocyte activation. Notably, RvD_n-3_ DPA display different potencies at regulating peripheral blood phagocyte and platelet activation, with RvD5_n-3_ DPA exhibiting the greatest ability to regulate these biological processes [164]. Recent studies indicate that the biological activities elicited by RvD5_n-3_ DPA on phagocytes are mediated by the orphan receptor GPR101 [166]. This receptor is expressed on human and mouse neutrophils, monocytes and macrophages and is activated by RvD5_n-3 DPA_ at nM – pM concentrations.

The biological activities of RvD5_n-3_ DPA are not limited to the vasculature. Investigations into mechanisms that contribute to the exacerbation of arthritic inflammation by the pathobiont *Porphyromonas gingivals* highlight a decrease in the concentrations of this mediator in the intestines of arthritic mice [167]. This decrease was linked with a disruption in intestinal barrier function, facilitating barrier breach and the consequent exacerbation of joint disease by *Porphyromonas gingivalis* [167]. Notably, administration of this mediator restores both barrier function and reduces arthritic inflammation. Zhou and colleagues recently described role for RvD5_n-3 DPA_ in experimental sepsis, whereby they observed that soluble fibrinogen-like protein 2 regulates the production of RvD5_n-3 DPA_ during experimental sepsis [168]. RvD5_n-3 DPA_ is also suggested to mediate the pro-resolving activities of the anticoagulant dabigatran [169].

Assessment of mechanisms that become activated early on during self-limited inflammation to fine tune phagocyte responses uncovered a novel family of autacoids produced in the circulation during interaction between vascular endothelial cells and neutrophils [163]. These mediators, termed 13-series resolvins, limit neutrophil trafficking to the site of infection, promote the uptake and killing of bacteria by phagocytes and the uptake of apoptotic cells by macrophages. These autacoids also counter regulate the production of pro-inflammatory eicosanoids, including leukotriene B_4_ and prostaglandins. They also downregulate the expression of caspase-1 and its pro-inflammatory product interleukin-1b in macrophages. Intriguingly the production of these immunomodulatory autacoids is upregulated by statins, with atorvastatin and pravastatin displaying the greatest propensity to increase the production of these mediators via the nitrosylation of COX2, which increases the catalytic activity of this enzyme [163,170]. This mechanism was found to be relevant in reducing inflammation during both infectious and sterile inflammation, suggesting that 13-series resolvins may be useful predictive functional biomarkers in evaluating the efficacy of statins at limiting inflammation [163,170].

Inflammation is now recognized to play a role in the pathophysiology of epilepsy. Lipid mediator profiling analysis of murine hippo-campi obtained from mice during experimental epilepsy identified a role for the n-3 protectin D1 (PD1_n-3 DPA_) in limiting disease severity [171]. Indeed, this mediator was found to be upregulated in epileptic mice. When mice were treated with PD1_n-3 DPA_ using a therapeutic paradigm, disease severity, including the expression of pro-inflammatory cytokines and the frequency and duration of epileptic seizures, was significantly reduced [171]. Furthermore, recent studies demonstrate that PD1_n-3 DPA_ is also able to increase the inhibitory drive onto the perisomatic region of the pyramidal neurons thereby limiting neuronal excitability [172].

#### 4.3.2. DPAn-3-derived SPM orchestrate leukocyte differentiation

In addition, to orchestrating host immune responses, DPAn-3 derived SPMs play a role in leukocyte differentiation. RvD5_n-3 DPA_ was recently found to contribute to T_reg_ differentiation from naive CD4+ T-cells. Temporal evaluation of lipid mediator profiles produced by differentiating T-cells revealed that this autacoid was upregulated during the early stages of T_reg_ differentiation. Furthermore, incubation of naive T-cells with RvD5_n-3_ DPA rescued the functional responses of Tregs differentiated in the presence of an ALOX15 inhibitor [173]. The DPA_n-3_-derived protectins (PD_n-3 DPA_), namely PD1_n-3 DPA_ and PD2_n-3 DPA_, were recently observed to coordinate monocyte-to-macrophage differentiation. Whereby, incubation of monocytes deficient in ALOX15 activity, the initiating enzyme in the PD_n-3 DPA_ biosynthetic pathway, with PD1_n-3 DPA_ or the PD_n-3 DPA_ biosynthetic intermediate 16S, 17S-epoxy-PD_n-3 DPA_ rectified monocyte-derived macrophage phenotype and their ability to uptake apoptotic cells [174].

#### 4.3.3. Upregulation of DPAn-3-derived SPM using dietary supplementation in humans

Studies evaluating approaches to upregulate endogenous SPM production demonstrate that the endogenous production of DPAn-3 derived SPM can be modulated following essential fatty acid supplementation. For example, Markworth and colleagues found that supplementation of healthy volunteers with DPAn-3 significantly increases RvD5_n-3 DPA_ [175]. This modulation of DPAn-3 derived SPM was also observed when healthy volunteers and patients with peripheral artery disease were administered an enriched marine oil supplement [176,177]. Intriguingly these changes, together with the upregulation of SPM from the DHA and EPA metabolomes, were linked with a regulation of peripheral blood phagocyte function [176,177]. Thus, results from these studies suggest that functional modulation of SPM via essential fatty acid supplementation may be linked with decreased circulating phagocyte activation and potentially a downregulation of inflammation.

### 4.4. Elovanoids

In the following section N.G. Bazan details the discovery of the elovanoids (ELVs) and identification of their mechanisms of action.

The significance of polyunsaturated fatty acids (PUFAs) has evolved from the broad concepts of providing membrane structural plasticity and fluidity for proteins diffusion and rotation to a diverse universe of functions. For example, DHA is necessary for sight, and when administered, is beneficial in x-linked retinitis pigmentosa and other neurode-generative diseases [178]. DHA from the diet is packaged by the liver and targeted to the central nervous system (CNS), where it achieves the highest concentration in photoreceptors and synaptic membranes [179].

PUFAs, precursors of lipid mediators and components of membrane lipids, comprise a new multidisciplinary field at the boundary of biophysics, chemical biology, and molecular physiology. Thus, at least two important issues have emerged: the gene that encodes the enzyme that elongates PUFAs to chain length ≥ 28 carbons (ELOVL4) is critically important for cell function, and their products are precursors of the new family of lipid mediators, the elovanoids (ELVs).

#### 4.4.1. ELOVL4

ELOVL4 catalyzes the rate-limiting condensation reaction for the synthesis of very long chain -saturated fatty acids (VLC-SFAs) and VLCPUFAs (chain length ≥ 28 carbons) [180]. This enzyme is expressed in brain neurons, photoreceptor cells, skin, testes, and meibomian glands [180]. In the skin, VLC-SFAs are components of sphingolipids, and these VLC-SFAs are necessary as a skin-permeability barrier [181]. ELOVL4 is selectively expressed in neurons and is evolutionarily conserved [182]. In photoreceptor cells, VLC-PUFAs are in phosphatidylcholines (PC) of the outer segment membranes, tightly bound to rhodopsin [183].

Mutation, loss, or downregulation of ELOVL4 is linked to retinal degeneration. Studies of a large familial group with retinal degeneration revealed an autosomal dominant macular dystrophy phenotype which results from a 5-bp deletion, causing Stargardt-like macular dystrophy [184,185], and an STGD3 mouse Elovl4 mutation produces a C32-C36 PC deficiency [186], leading to the suggestion that loss or reduced VLC-PUFAs may cause loss of photoreceptors or functional perturbations [187], highlighting the importance of these molecules in the retina. Therefore, because of the inability to take up and incorporate DHA and the absence of VLC-PUFAs in the degenerating adiponectin receptor 1 (AdipoR1)^−/−^ mouse retina, the synthesis of these molecules must rely on the presence of DHA. The occurrence of central geographic atrophy (CGA) and neovascular age-related macular degeneration (AMD) was found to be 30% less likely with high omega-3 LC-PUFA (e.g., DHA) intake [188], emphasizing the importance of maintaining adequate dietary amounts of DHA for retinal homeostasis.

Neuron-specific ELOVL4 is expressed in the CNS, including in hippocampal neurons of the dentate gyrus (DG) subgranular layer, a locus for medial temporal lobe epilepsy. Mutations in ELOVL4 lead to impaired neural development, mental retardation, neuronal dysfunction, hyperexcitability, and seizures [189].

#### 4.4.2. Elovanoids are a new class of bioactive lipids synthesized from C32 or C34 FA precursors

**Fig. 5.**
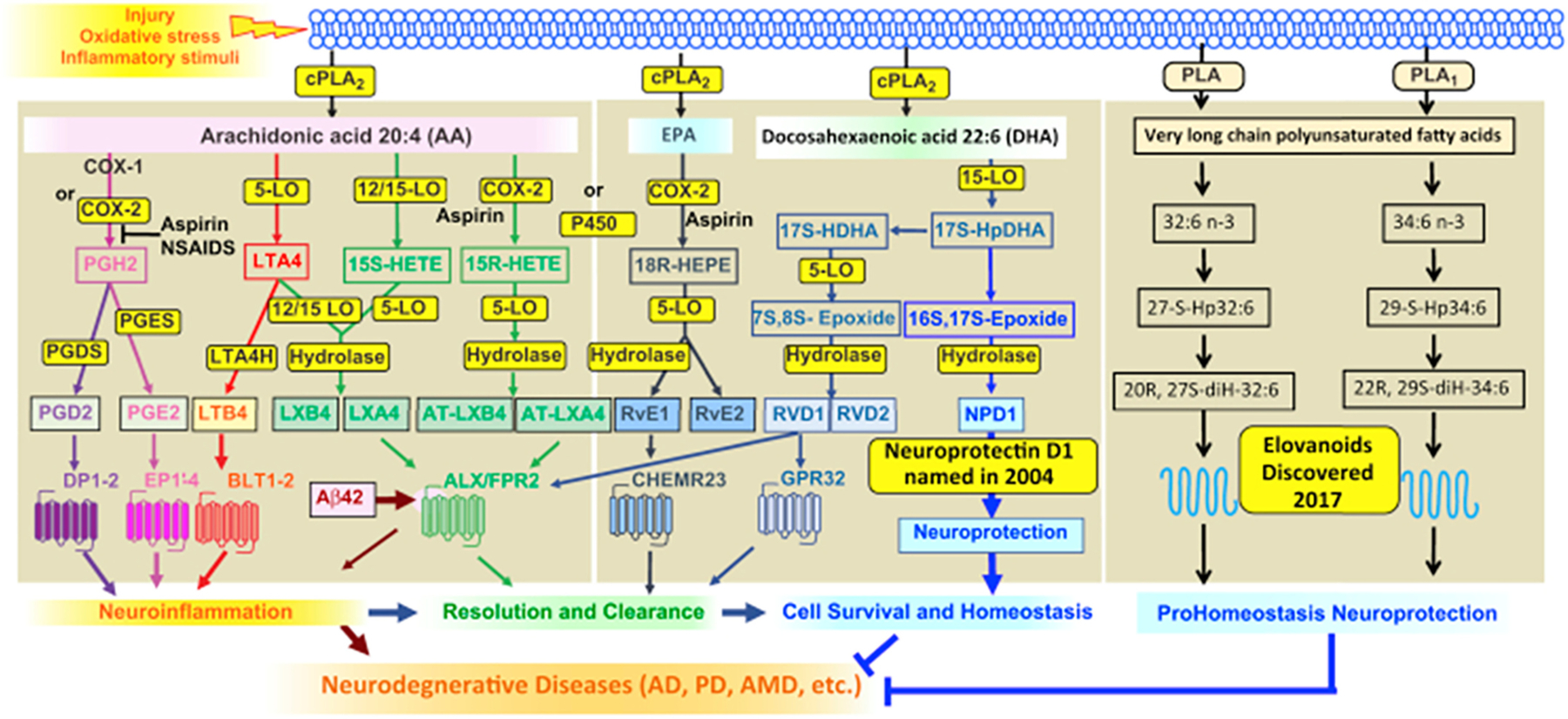
Eicosanoids, docosanoids, and elovanoids. PLAs that release ARA, EPA, DHA, or VLC-PUFAs are depicted at the top. Synthesis of mediators and receptors involved are illustrated. The outcome is modulation of inflammatory responses and homeostasis. AD, Alzheimer’s disease; AMD, age-related macular degeneration; VLC-PUFA, very long-chain PUFA. Reproduced, with permission from the *Journal of Lipid Research* [178].

**Fig. 6.**
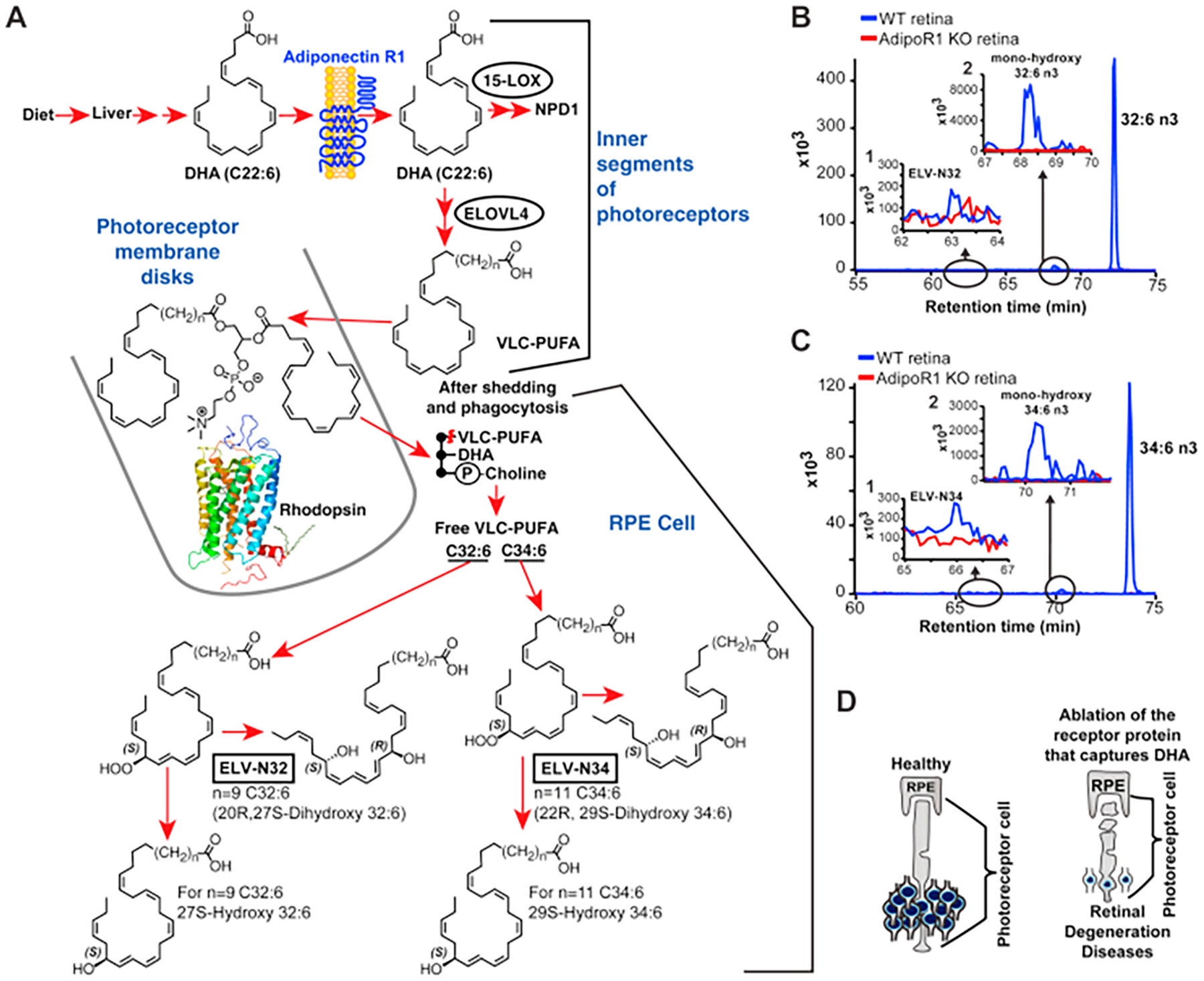
Genetic ablation of adiponectin receptor 1 leads to depletion of VLC-PUFAs and its derivatives in retina. A: Dietary DHA, or DHA derived from dietary 18:3n3, is supplied by the liver and taken by the Adiponectin Receptor 1 (AdipoR1), followed by elongation in the inner segment of photoreceptor cell (PRC) by Elongation of Very Long chain fatty acids-4 (ELOVL4) to VLC-PUFA and incorporation into PC molecular species, which contains DHA at sn-2. During daily PRC outer segment renewal, these PC molecular species interact with rhodopsin and, after shedding of the PRC tips and phagocytosis, become part of retinal pigment epithelium (RPE) cells. Uncompensated oxidative stress (UOS) or other disruptors of homeostasis trigger the release of VLC-PUFAs. 32:6n-3 and 34:6n-3 are depicted generating hydroperoxyl forms, and then elovanoid (ELV)-N32 or ELV-N34, respectively. B: The pool size of free 32:6n-3 in retinas of AdipoR1 KO mice (red) is decreased as compared with that in wild type (WT) (blue). Insert (1) shows ELV-N32 in KO (red) and WT (blue); insert (2) shows monohydroxy 32:6n3, the stable derivative of the hydroperoxyl precursor of ELV-N32, in WT (blue) and lack of detectable signal in the KO (red). C: Similarly, the pool size of free 34:6n-3 in retinas of AdipoR1 KO mice (red) is decreased as compared with that in WT (blue). Insert (1) shows ELV-N32 in KO (red) and WT (blue); insert (2) shows mono-hydroxy 34:6n-3, the stable derivative of the hydroperoxyl precursor of ELV-N34, in WT (blue) and lack of detectable signal in the KO (red). D: RPE cells sustain PRC functional integrity (left); right, the ablation of AdipoR1 switches off DHA availability, and PRC degeneration ensues. Reproduced, with permission, from *Scientific Reports* [190]. (For interpretation of the references to colour in this figure legend, the reader is referred to the web version of this article.)

In 2017, elovanoids (ELVs) were discovered and named [190–192]. This new class of endogenous lipid mediators is distinct from the widely known lipid mediators produced from PUFAs with C20 and C22, such as the classical eicosanoids and SPMs. ELV-N32 and ELV-N34 are stereo-specific di-hydroxylated derivatives of 32:6n-3 or 34:6n-3 (Fig. 5), respectively, made by the elongase ELOVL4 (elongation of VLC-FAs-4), which converts C26-derived FAs from EPA or DHA to VLC-PUFAs, ≥C28. These PUFAs are mainly esterified at the C1 (sn-1) position of PC that has DHA in the C2 (sn-2) position, and upon the appropriate stimulus (e. g., uncompensated oxidative stress), are released by phospholipase A1 (PLA1) and/or PLA2 for the formation of ELVs, NPD1, or other docosanoids (Fig. 6). Here, I describe key events in the discovery of ELVs and highlight some of their functions.

#### 4.4.3. The discovery of Elovanoids

In short, the discovery of ELVs was the product of curiosity, resulting in a driven jump of knowledge, not an incremental finding. In 2015, we reported that AdipoR1 genetic deletion leads to a shutting off of the uptake and retention of DHA that produced a cell-selective DHA lipidome-specific impairment in retinal pigment epithelium (RPE) cells and PRC function followed by PRC degeneration [193]. Unexpectedly, a molecular species of PC containing both VLC-PUFAs and DHA was depleted in the knockouts (KOs). Since this PC molecular species closely interacts with rhodopsin [194], one possibility for the PRC degeneration phenotype of the AdipoR1 KO was that its absence triggered PRC demise. An alternative hypothesis was that there was a shortage of some biologically active mediator derived from VLC-PUFAs.

To begin testing this hypothesis, we explored and found that the free VLC-PUFAs pool size was depleted in the RPE of the AdipoR1 KOs. Initially, our thinking was that this observation was expected because, as our lab had defined earlier, the recycling of DHA between these two cells takes place to retain/conserve DHA; we had called this the “short loop of recycling” [195–197]. Thus, the idea came from a separate, very different, biologically active derivative formed in the RPE cells from VLC-PUFA precursors. LC-MS/MS of wildtype (WT) and not in KO revealed in the RPE peaks that were not free VLC-PUFAs. Our initial marker was the absence of those peaks in our KO. When we collected them from extracts of several WT RPEs, we found initial UV evidence of hydroxylated VLC-PUFAs, and when we added them to RPE cells challenged by uncompensated oxidative stress (H_2_O_2_ plus TNF-α), cell survival was elicited. Since this happened preceding retinal degeneration, we speculated that they might play a role in the survival of RPE and PRC, therefore sustaining sight.

We fully characterized these novel lipids and defined the complete structures and stereochemistry of the novel elovanoids, ELV-N32 (derived from 32:6n3) and ELV-N34 (derived from 34:6n3), the complete R/S configuration, and the Z/E geometry of the double bonds as generated in retinal cells and neurons (Fig. 6) [190,191]. In 2019, we disclosed that ELVs sustain RPE and PRC integrity when confronted by injury via arresting the expression of senescence programs and other genes [198].

These findings are different from other endogenous prohomeostatic and neuroprotective mechanisms because they involve a phospholipid molecular species that is endowed with acyl chains with two different PUFA precursors of bioactive lipids. This unusual signaling encodes two PUFA-derived lipid mediators, the precursors of which are stored in specific PC molecular species. Whereas DHA, which is the first-described PUFA precursor of NPD1 [130,133,199], is located at the sn-2 position of the phospholipids, the VLC-PUFAs are located at the sn-1 position and are subject to alternatively or concomitantly regulated pathways (Fig. 6). Therefore, the findings revealed here feature a different signal bifurcation prohomeostatic and neuroprotective mechanism that aims to sustain neural cell integrity. Because there are fatty acids longer than 34:6n-3 and products of other ELOVL enzymes, we anticipate that other ELVs might also be endogenously made to regulate cell function.

#### 4.4.4. Elovanoids are neuroprotective in experimental ischemic stroke

We demonstrated that ELV-N32 or ELV-N34, when applied to cerebral-cortical mixed neuronal cells or hippocampal mixed neuronal cells in culture, can overcome the damaging effects of uncompensated oxidative stress or NMDA-induced neuronal excitotoxicity. Most of the strokes are ischemic in nature [200], and deprivation of oxygen and glucose leads to a cascade of events involving mitochondrial damage, which ultimately leads to neuronal death. Therefore, the *in vitro* oxygen-glucose deprivation (OGD) model provides an opportunity for teasing out the cellular events and putative underlying neuroprotective signaling pathways in which ELVs participate. We showed that both ELV-N32 and ELV-N34 elicit neuroprotection and overcome neuronal cytotoxicity. We also showed that the 34C omega-3 VLC-PUFA (C34:6n-3) precursor of ELVs, when applied at a dose of 250 nM after 2 h of reoxygenation phase following 90 min of OGD insult, could provide neuroprotection to cerebral-cortical neurons. In conclusion, the endogenously generated ELVs (ELV-N32 or ELV-N34) ameliorated neuronal injury induced by several stressors, such as NMDA receptor activation, uncompensated oxidative stress, or OGD in cerebral-cortical mixed neuronal and hippocampal mixed neuronal cultures.

Next, we showed that ELV treatments delivered at 1 h after 2 h of experimental ischemic stroke improved neurological recovery throughout the 7-day survival period. We also used magnetic resonance imaging (MRI), a highly sensitive tool for the detection of changes in water content and diffusion, both of which characterize acute ischemic stroke [201]. The rapid induction of brain edema following focal ischemia is the leading cause of morbidity and death after stroke [202]. Maximum protection was detected in the cortex (the penumbral area) and also in the subcortical area. Histopathology revealed smaller infarcts in cortical and subcortical areas with less pancellular damage, denser eosinophilic areas, and shrunken neurons along the infarct margin, all of which were detected in ELV-treated rats.

Cerebral ischemia initiates a complex cascade of cellular, molecular, and metabolic events that lead to irreversible brain damage [203]. Dead neurons and injured tissue are scavenged by activated resident microglia and/or macrophages that invade the injured tissue from the bloodstream. Surviving astrocytes and activated microglia in the penumbra may facilitate restoration of neuronal integrity by producing growth factors, cytokines, and extracellular matrix molecules involved in repair mechanisms [204]. Our results demonstrated that ELV treatment increased the number of NeuN-positive neurons and GFAP-positive reactive astrocytes and the SMI-71-positive blood vessel density in the cortex [191]. Blood vessel integrity facilitates neurogenesis and synaptogenesis, which, in turn, contribute to improved functional recovery. We showed here that the newly identified ELVs protected neurons undergoing OGD or NMDA receptor-mediated excitotoxicity. Moreover, ELVs attenuated infarct volumes, rescued the ischemic core and penumbra, diminished NVU damage, and promoted cell survival accompanied by neurological/behavioral recovery. It is reasonable to propose that novel ELV therapies have the potential to treat focal ischemic stroke and other conditions that engage inflammatory/homeostatic disruptions.

#### 4.4.5. Mechanism of action of Elovanoids

**Fig. 7.**
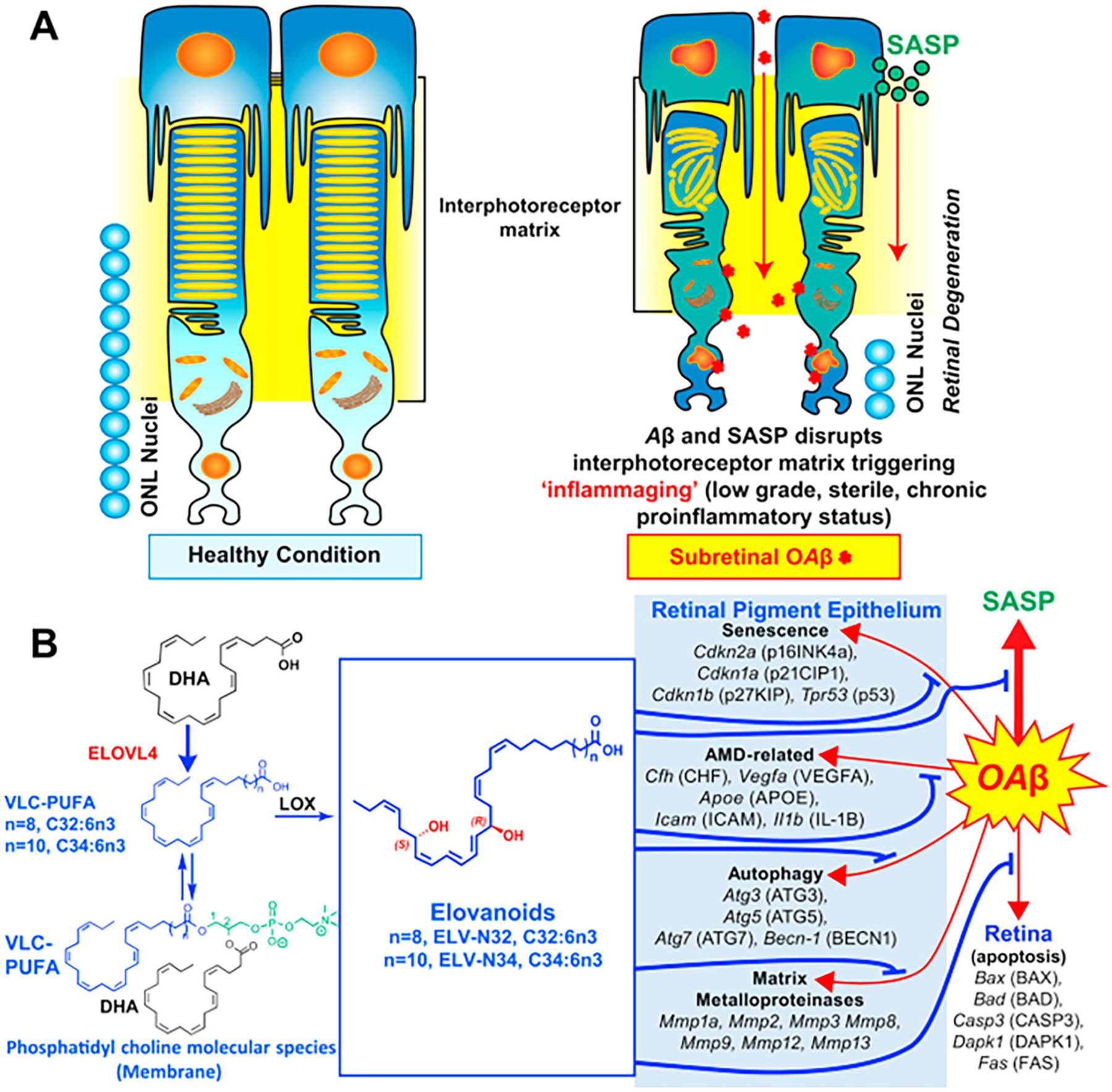
ELVs effect on oligomeric amyloid-β (OAβ)-induced RPE and PRC damage. A: OAβ induces a senescence gene program and disrupts RPE tight junctions. OAβ penetrates the retina, causing PRC cell death in our in vivo WT mice study, as reflected in less outer nuclear layer (ONL) nuclei (Fig. 5 from [198]). OAβ activates the senescence-associated secretome (SASP) that contributes to perturbing the interphotoreceptor matrix (IPM), triggering inflammaging in PRC and also likely in Mueller glia, which limits the IPM. Therefore, senescence paracrine expression takes place. ELVs restore RPE morphology and PRC integrity. B: OAβ induces expression of senescence, autophagy, matrix metalloproteinases, and age-related macular degeneration (AMD)-related genes in the RPE and apoptosis genes in retina in addition to p16INK4a protein abundance. ELVs downregulated the OAβ-gene inductions and p16INK4a protein abundance. Pathways for the ELV synthesis are outlined. ELV, elovanoid; PRC, photoreceptor cell; RPE, retinal pigment epithelium. Reproduced, with permission, from the *Proceedings of the National Academy of Sciences of the United States* [198].

In so far as the mechanism of action, ELVs target the expression of protective proteins and behaves as senolytic (Fig. 7). ELVs counteracted the cytotoxicity of OAβ subretinally administered in WT mice leading to RPE tight junction disruptions followed by PRC cell death. Our data show that OAβ activates a senescence program reflected by enhanced gene expression of *Cdkn2a*, *Mmp1a*, *Trp53*, *Cdkn1a*, *Cdkn1b*, Il-6, and senescence-associated secretory phenotype (SASP) secretome, followed by RPE and PRC demise (Fig. 7), and that ELV-N32 and ELV-N34 blunt these events and elicit protection to both cells. P16INK4a protein abundance is also targeted. The RPE cell is terminally differentiated and originated from the neuroepithelium. In this connection, senescent neurons in aged mice and models of Alzheimer’s disease [205] and astrocytes [206] also express senescence and develop secretory SASP that fuels neuroinflammation in nearby cells [207]. This is likely the case in our study reported in 2019 [198], where neighboring cells may be targeted by SASP neurotoxic actions, inducing photoreceptor paracrine senescence. Therefore, SASP from RPE cells may be autocrine and paracrine, altering the homeostasis of the interphotoreceptor matrix microenvironment (Fig. 7), as a consequence and creating an inflammatory milieu that contributes to loss of function associated with aging, age-related pathologies [208], Alzheimer’s disease, and likely AMD. Furthermore, ELVs restore expression of ECM remodeling matrix metalloproteinases altered by OAβ treatment, pointing to an additional disturbance in the interphotoreceptor matrix. The inflammation set in motion may be a low-grade, sterile, chronic proinflammatory condition similar to inflammaging that is also linked to senescence of the immune system [208,209]. In addition, ELVs counteracted OAβ-induced expression of genes engaged in AMD and autophagy. It remains to be defined whether the ELVs targeted events on gene transcription (Fig. 7) to inform novel unifying regulatory mechanisms to sustain health span during aging and neurodegenerative diseases [208,210]. Several forms of retinal degenerative diseases, including retinitis pigmentosa and other inherited retinal degenerations, may underlie these mechanisms, and ELVs might halt the onset or slow down disease progression. Although further research is needed, our results, overall, show the potential of ELVs as a possible therapeutic avenue of exploration for neurodegenerative diseases.

## 5. Non-enzymatically oxidised-PUFAs (NEO-PUFAs)

In the following section T. Durand and J.-M. Galano describe the biosynthesis, structures and activities of nonenzymatically derived PUFAs (NEO-PUFAs).

### 5.1. Lipid peroxidation

Lipid peroxidation (LPO) is a degenerative process implicated in the pathogenesis of diseases and/or involved in the resolving process of diseases by the production of signaling molecules or lipid mediators. It is also a very common process in the plant kingdom and invertebrates, which is outside of the scope of this review, and interested readers should refer to the following recent review [211].

The process of nonenzymatic peroxidation of PUFAs, which is exacerbated under oxidative stress (OS) conditions, produces a myriad of oxidized compounds, some structurally similar to the oxylipins (racemic PGF_2α_) or structurally unique (as not represented in the enzymatic process; i.e., isofurans or isomeric series of prostaglandins, the isoprostanes). We have recently tentatively abbreviated them as NEO-PUFAs, i.e., nonenzymatic oxygenated PUFAs to differentiate them from the enzymatically-derived oxylipins [212]. The NEO-PUFAs are part of the redox-lipidome, and while they have been investigated as biomarkers of diseases (the most frequently investigated are the isoprostanoids) [213], they are however rarely considered biologically relevant molecules [214].

### 5.2. Mechanisms of formation of cyclic NEO-PUFAs

The free radical nonenzymatic oxidation of PUFAs has been studied for more than 70 years in biology and medicine, in parallel with the study of oxidative stress. At the beginning of the 1990s it became evident that limitations were inherent in the exploration of OS and LPO *in vivo* [215]; however, a seminal paper appeared with the potential of solving these limitations. Morrow and co-workers showed that mass spectrometry could detect and quantify prostaglandin-like compounds *in vivo* in plasma and tissues, and these compounds were named Isoprostanes (IsoP) [216]. They pinpointed that a nonenzymatically driven biosynthetic process led to the generation of compounds structurally similar to the enzymatically-derived prostaglandins, but with a much greater diversity of isomers, for example, compared to the single enzymaticaly derived prostaglandin (PGF_2α_) from ARA, there are four different types of IsoPs. Not long afterwards, the isoprostanes were shown to be ubiquitous in human fluids and tissues and fairly easy to quantify, which led to them becoming the long-sought after gold standard biomarker of oxidative status of free radical injuries in humans [217].

Many other NEO-PUFAs have subsequently been identified, some similar to their enzymatic version (albeit racemic and with the full set of potential isomers), like the isolevuglandin, isotromboxane, but also a novel structure was discovered which is unique to nonenzymatic biosynthesis, the isofuranes.

**Fig. 8.**
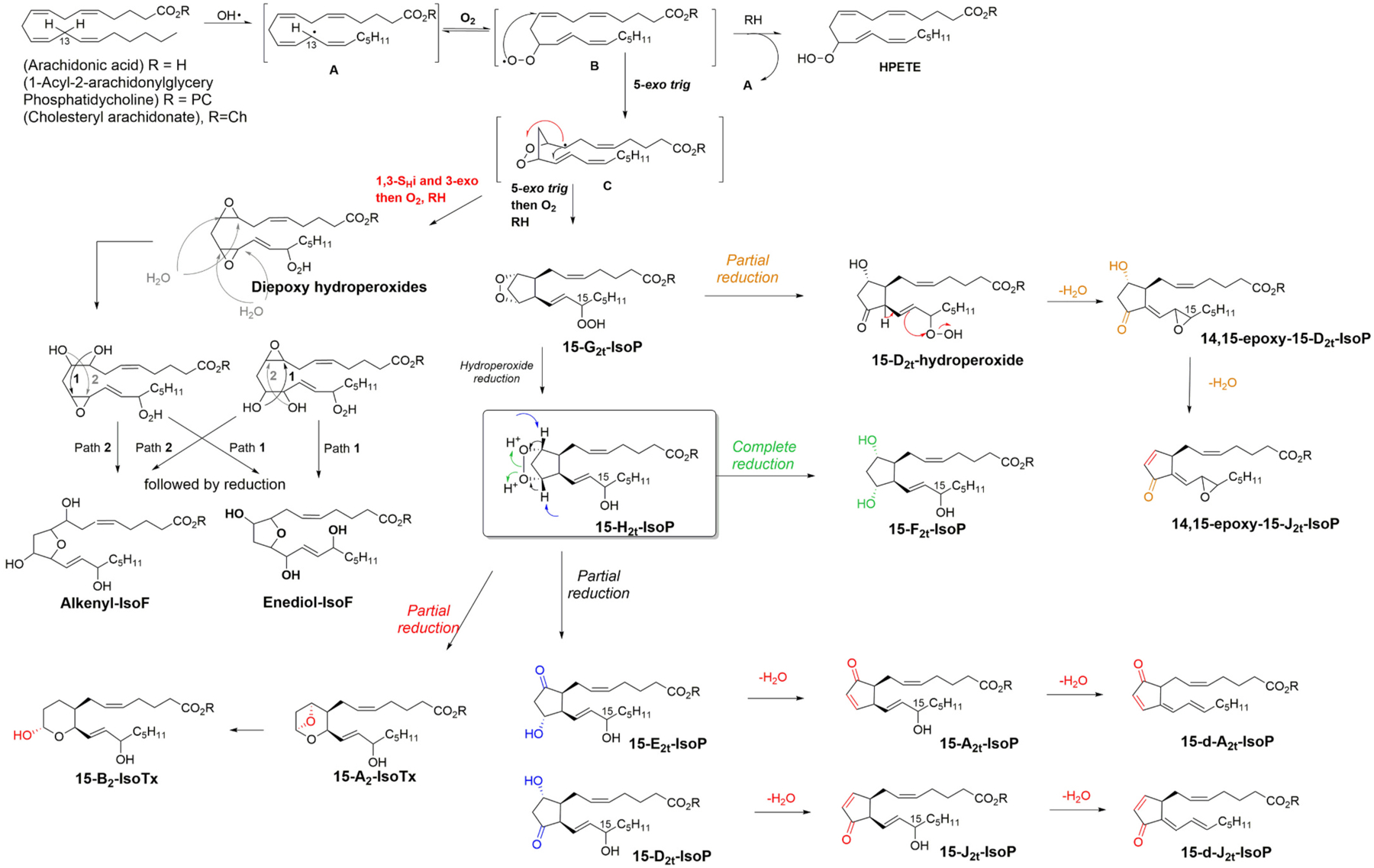
Mechanism of the free radical chain process leading to cyclic NEO-PUFAs. (Only one H-atom abstraction is shown for clarity, as well as stereoisomers, and ARA was chosen as the PUFA). See text for details.

Fig. 8 shows the formation of IsoPs from ARA (for simplicity only one the four possible series of IsoPs is shown), which starts with H-atom abstraction at the 13th position of the ARA ester. Unlike the enzymatic process of oxylipin formation, the free radical chain process occurs to membrane-bounded PUFAs. All that is required to make cyclic NEOPUFAs is a PUFA with at least three double-bonds separated by a methylene group.

The initial step in the free radical chain peroxidation process starts by an initiation step, that is the initial formation of a free radical (like the hydroxyl radical (HO^●^) one of the oxygen center radicals overproduced under oxidative stress conditions), which will abstract a H atom from a PUFA at the origin of the pentadienyl radical **A** (Fig. 8) [218]. Then only, the radical chain process can produce hydroperoxides (hydro-peroxyeicosatetraenoic acids; HPETEs) via O_2_ addition and hydro-peroxyl radicals and (**B**). The propagation can be maintained indefinitely in theory until termination step mechanisms. However, other propagation mechanisms are also in competition, like a peroxyl radical cyclisation (via a 5-exo trig process) to give 1,2-dioxolanylalkyl radicals (**C**), which has several subsequent mechanistic fates, and two of them lead to cyclic NEO-PUFAs [219], which are detailed below.

The first one starts with a second 5-exo trig cyclization onto the conjugate diene leading after oxygen trapping and H atom abstraction to IsoP substructure G_2t_-IsoP. Of particular interest here are the production of epimers of prostaglandins, as the lipid lateral chains generated can be of 1,2-*cis* configuration or 1,2-*trans* configuration (only the 1,2-*cis* configuration is represented in Fig. 8), and the *cis* or *trans* configuration can be anti or syn to the always fixed 1,3-*cis* diol (because of endoper-oxide production)). A perfect match with PGF_2α_ is also produced via this mechanism (albeit racemic), and Morrow and co-workers highlighted the issues with PGF_2α_ quantification in urine [220]. This makes four different isomers for the four consecutive centers, plus the epimeric center at the allylic position, so a total of eight possible stereoisomers for one single series (hence the sheer complexity of NEO-PUFAs compared to enzymatically derived oxylipins). Another fate of **C** (Fig. 8) is by 1,3 S_H_*i* followed by a 3-exo trig cyclization to give rise to diepoxy hydro-peroxides after O_2_ trapping and H atom abstraction [221].

G_2_-IsoP is partially reduced into H_2_-IsoP and its complete reduction affords the F-type of isoprostane [216]. Partial reductions depending on the tissues and/or pathophysiological conditions can make ketohydroxy IsoPs (Fig. 8, blue structures) E_2_-IsoP or D_2_-IsoP [222] (it has been showed that E and D-IsoP can be epimerized into their corresponding prostaglandins under physiological conditions) [223]. Dehydration of membrane bound E_2_- and D_2_-IsoPs is also feasible in physiological conditions and cyclopentenone A_2_ and J_2_ were described as very reactive intermediates [224]. They also tend to deoxygenate further into deoxy-J_2_-IsoP to even more reactive biomolecules [225]. Interestingly deoxy-A_2_-IsoP has not thus far been reported in the literature; however, based on the mechanism identified all the series of J_2_-IsoP it suggests deoxy-A_2_-IsoPs must exist.

Another partial reduction can lead to a thromboxane substructure, and while A_2_-IsoTX could not be detected; B_2_-IsoTx was found in CCl_4_ induced injury of rats. *In vitro* oxidation of ARA initiated by Fe/ADP/ascorbate also led to B_2_-IsoTx [226]. The epoxy-IsoPs can only be explained by the unique rearrangement-elimination sequence of 15-D_2_-hydroperoxides or 15-E_2_-hydroperoxides (not represented here), which originate from endoperoxide G_2_-IsoPs [227]. Such 14,15-epoxy-15-D_2_-IsoP as described can also further dehydrate to 14,15-epoxy-15-J_2_-IsoP [228]. Another cyclic NEO-PUFAs unique from the nonenzymatic autooxidation of lipids are the isofuranes (IsoF) [229], which are best described biosynthetically from diepoxy hydroperoxides [219]. Such bisepoxides can react with water to ring open one of the epoxides, and the resulting hydroxyl group will then attack the remaining epoxide to form the furan cycle. Two types of IsoF were named as alkenyl-IsoF and enediol-IsoF depending of the nature of the side chains. The final stage of biosynthesis is the release of the oxidized PUFAs from the membrane, which occurs via the action of phospholipase A_2_.

### 5.3. Biological relevance of cyclic NEO-PUFAs

Since the 1990’s 15-F_2t_-IsoP and other derivatives have been commonly used as the best available standard for measuring the extent of lipid peroxidation in most tissue fluids. However, these cyclic NEO-PUFAs are also relevant to human pathologies, due to their harmful or beneficial actions produced via their activities at prostanoid and ryanodine receptors.

The initial report by Morrow and co-workers identified levels of 15-F_2t_-IsoP in plasma were one or two orders of magnitude higher than PGF_2_, and 15-F_2t_-IsoP was an extremely potent renal vasoconstrictor in the low nM range [216]. Since then, investigations have revealed its vasoconstrictive effects in many vascular beds (heart, liver, lung, kidney, smooth muscle, retina) via the thromboxane receptor (TP) [230]. 15-F_2t_-IsoP can also modulate platelet activity via the same receptor [231]. 15-E_2t_-IsoP is also a vasoconstrictor via the TP and the dichotomy of PGF_2α_ vs PGE_2_ (vasoconstrictor vs. vasodilator) was not observed [232]. 15-E_2t_-IsoP is also a ligand of E-series of prostaglandin receptors (EP) [233], and bronchoconstrictor in lung. Bendorf and co-workers further showed that 15-F_2t_-IsoP, 15-E_2t_-IsoP and 15-A_2t_-IsoP inhibited the VEGF-induced migration and tube formation of endothelial cells, and that altogether inhibit angiogenesis via activation of the TBXA2R [234]. 15-J_2_-IsoP was found to have inflammatory response by inhibiting via the peroxisome proliferator-activated receptor gamma (PPARγ) activation and induce RAW264.7 cell apoptosis in a PPARγ-independent manner [235]. 15-A_2_-IsoP another cyclopentenone ARA derivative displays anti-inflammatory effects by the inhibition of NF-κB pathway in lipopolysaccharide (LPS)-induced macrophages and human gestational tissues [236]. The overall picture of these cyclopentenone metabolites is currently unclear, as recently highlighted for their prostaglandin equivalents, and is probably much dependent on their structures [237]. For example, 1-palmitoyl-2-(5,6-epoxyisoprostane E_2_)-sn-glycero-3-phosphocholine, (PEIPC) in OxPAC (oxidized 1-palmitoyl-2-arachidonoyl-*syn*-glycero-3-phosphocholine) was shown to regulate over 80% of the 1000 genes regulated by OxPAC in human aortic endothelial cells (HAEC), and their non-esterified epoxy-IsoPs possess similar functions on genes showing a dual and opposing bioactivity in inflammation, depending on their concentration or their substructures [237,238].

There is little information available for EPA cyclic NEO-PUFAs in the literature, partially due to a lack of commercial standards. Synthetically available 5-F_3t_-IsoP was found to behave in a similar manner to other F_2_-IsoPs in modulating the release of neurotransmitters in isolated bovine retina via prostanoid receptors [239], while cyclopentenone 15-A_3t_-IsoP had anti-inflammatory effects on LPS-stimulated macrophages, via the inhibition of NF-κB pathways, and inhibitory effect on the formation of foam cells, a major step in the pathogenesis of atherosclerosis [240].

DHA cyclic NEO-PUFAs have been investigated to a greater extent than the EPA derivatives, as there has been a greater focus on neuroprostane synthesis. 14-A_4_-NeuroP is a potent anti-inflammatory mediator, inhibiting NF-κB activation in LPS-induced macrophages [241], Majkova et al., showed that A_4_/J_4_-NeuroPs down-regulated PCB77-induced monocyte chemo-attractant protein-1 expression and nuclear factor erythroid 2-related factor 2 (Nrf2) activation in primary pulmonary endothelial cells [242], and Gladine and co-workers showed that both 4-(RS)-4-F_4t_-NeuroP and 14-A_4t_-NeuroP displayed anti-inflammatory activities, similar to the protectins in human macrophages. These results can parallel the study that F_4_-Neuroprostanes as the best oxylipin-NEO PUFA predictor of atherosclerosis in atherosclerosis prone mice, which received increasing doses of DHA [243]. One particular focus of our group is the biological relevance of 4-(RS)-4-F_4t_-NeuroP and its unique ability to protect the ryanodine receptors *in vitro* and *in vivo*, where potent antiarrhythmic properties have been shown via this mechanism of action [244]. Recently, F_4_-NeuroPs showed a biological activity in sperm function and was able to induce capacitation via increasing AMPK phosphorylation, and its role at ryanodine receptors is currently being explored [245]. Finally, Lee and co-workers showed that 4-(RS)-4-F_4t_-NeuroP can cross the blood brain barrier into rat brain tissue and alter brain omega-3 and omega-6 PUFA profiles, where anti-inflammatory and pro-resolvin lipid biomarkers were significantly elevated [246]. Furthermore, 4-F_4t_-NeuroP treatment to human neuroblastoma cells and rat primary neuronal cells consistently elevated HO-1 mRNA expression, suggesting that native 4-F_4t_-NeuroP has a regulatory role in neurons for cell survival [247].

Cyclic NEO-PUFAs and particularly the IsoPs have been detected and quantified from the most important PUFAs. Their biosynthesis is well known, their quantification is straightforward, and their biological relevance should grant them a place next to the well-known mediators of diseases. However, it took three decades for the field of classical eicosanoids and other oxylipins to begin to be recognized clinically. A recent paper provides the way forward to raise awareness of the oxylipins and NEO-PUFAs in clinical settings [246].

## 6. Fatty acid esters of hydroxy fatty acids

In the following section L. Balas presents and overview of the recently identified family of branched fatty acids, the fatty acid esters of hydroxy fatty acids (FAHFAs).

In contrast to PUFAs, saturated fatty acids are generally thought to be deleterious to health, promoting cardiovascular diseases, obesity, and type 2 diabetes. Thus, the paradoxical and striking discovery of endogenous saturated anti-diabetic acyloxy fatty acids, called fatty acid esters of hydroxy fatty acids (FAHFAs) [248] triggered a strong revival of interest in these lipids. Structurally, these lipokines are characterized by a branched ester linkage between a fatty acid (FA) and a hydroxy-fatty acid (HFA). Nowadays, the term “branched” is often omitted, although it is an important aspect. A brief overview is presented below, including non-branched isomers and functionalized polar head derivatives.

### 6.1. Branched FAHFAs

Hundreds of structures with saturated, monounsaturated, or PUFA chains, including regio-isomers with the ester linkage at position C5 or C7 to C13 and their (R)- and (S)-epimers have been identified [248,249]. Although present in some natural products (see below), the 3-series does appear to be part of this anti-diabetic lipid family.

Branched FAHFAs are endogenously produced in insects [250], and mammals, such as rodents [248,251], caribou and moose [252], and humans [251,253]. In humans, white adipose tissue (WAT) represents the major site of FAHFA synthesis [248,253–255], although they are also found in blood [256,257] and other tissues, such as the liver [248,258], kidneys [248,258,259], large intestine [260], pancreas [248], lungs [258], thymus [258], and heart [258], albeit to a lesser extent. Branched FAHFAs are also naturally occurring substances found in microalgae [261], breast milk [262] and foods, such as cereals, fruits, vegetables, oils, eggs and meat [251,263–265]. Quantities are rather low, ranging from 45 to 320 ng/g of fresh food. To date, no information has been reported about their absorption and bioavailability.

Since their discovery, only a few research groups have begun to investigate their biosynthetic pathways, and roles in health and diseases, and of the hundreds of possible FAHFA structures very few have been studied thus far. For example, little is known about the biosynthesis of saturated hydroxylated fatty acids [249], although Kuda and co-workers reported in 2018 that 9-hydroxylated stearic acid is produced from (per) oxidized membrane phospholipids [266]; however, the regioselectivity of hydroxylation/peroxidation on some carbon atoms of the fatty acid chain (positions 5,7, 9, 10, 11, 12, 13 mainly) has yet to be explained. The advent of synthetic standards should facilitate research, and their preparation has been summarized in a recent comprehensive review [267].

It is possible that FAHFAs do not all have the same properties nor the same intensities in their effects. The palmitic acid hydroxy stearic acid (PAHSA) family is currently the most studied, showing significant enhancement of glucose tolerance, glucagon-like peptide 1 secretion and insulin sensitivity in obese insulin-resistant mice with reduction of the adipose tissue inflammation [248,249,268,269]. PAHSA concentrations inversely correlate with insulin resistance and the propensity to develop diabetes. 9-PAHPA (palmitic acid esterified to 9-hydroxy palmitic acid) and 9-OAHPA (oleic acid esterified to 9-hydroxy palmitic acid) increase insulin sensitivity in obese and healthy mice and they both increase basal metabolism [270,271]. Effects of human blood 9-PAHSA and 9-OAHSA suggest a protection against cardiovascular diseases [256]. In both mice [251,272] and humans [257], polyunsaturated FAHFAs exert powerful anti-inflammatory properties, stronger than the fully saturated compounds. The beneficial effects of branched FAHFAs, namely involvement in metabolic disorders and diabetes, inflammation, browning of WAT, potential antioxidant and anti-cancer properties and the current knowledge on their biosynthesis and metabolism are summarized in recent reviews [249,273]. In addition, recent investigations have shown noticeable decreases in FAHFA levels in plasma of patients suffering from acute coronary syndrome or acute ischemic stroke [257], and increasing levels with the severity of lupus nephritis [270] in murine models.

### 6.2. The 2-FAHFA series

Levels of anti-inflammatory 2-FAHFAs with very short FA chains (C2-C5) increase in the colon of influenza infected mice compared to healthy controls [274].

### 6.3. Non-branched FAHFAs (omega-FAHFAs)

Mainly studied in meibonian glands and tear film of human and mice eyes [275,276], these omega-FAHFAs are also present in equine amniotic fluid [277], mice skin [278], vernix caseosa [279], and equine sperm [280]. With their long or very-long (C16 to C38 atoms) chains and their carboxylated acid group, these amphiphilic lipids (ca. 4% total lipid) promote tear film stability and prevent drying of the ocular surface [276,281,282]. Cholesteryl esters of omega-FAHFAs have also been reported [279].

### 6.4. Functionalized polar head FAHFA derivatives

#### 6.4.1. Triacylglycerol-estolides (TAG-Est)

*In vivo* esterification of FAHFAs with diacylglycerol produces TAG-Est that serves as reservoirs of FAHFAs. In mice, the fine-tuned TAG-Est metabolism (liberating free FAs or free FAHFAs) regulates the anti-diabetic signaling lipid profiles [254,283,284]. In oat, a digalactosyldiacylglycerol containing 15-LAHLA in place of a FA chain has been reported [285].

#### 6.4.2. Amino acid-containing FAHFAs

**Fig. 9.**
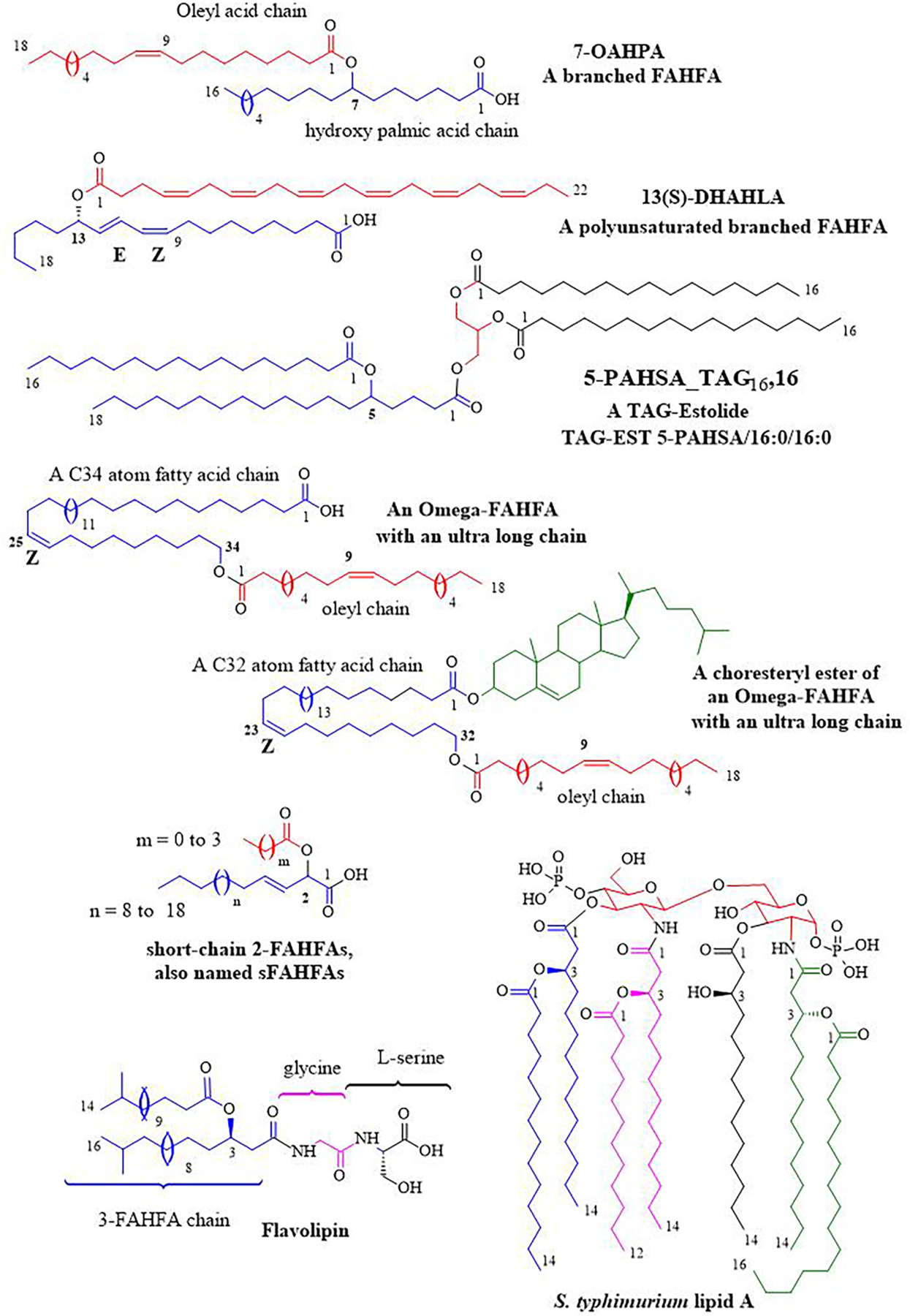
Examples of Fatty acid esters of hydroxy fatty acids (FAHFAs) and derivatives. See text for details.

Several amino acid-containing acyloxyacyl lipids have been reported. Structurally (Fig. 9), they are composed of a 3-FAHFA bound to the amine group of an amino acid(lysine [286], glycine [287], ornithine [288,289], di- and tri-methylated ornithine [290]) or dipeptide such as in flavolipin (a serine-glycine polar head and a omega-1 methyl group on both fatty chains)[267] or cerilipin [286,291].

They are essentially found in the outer membranes of Gram-negative bacteria and also in some Gram-positive bacteria. In 2010, Geiger published a review dedicated to these amino-acid lipids [292]. More recently, an anti-bacterial activity against *Streptococcus agalactiae* and a cytotoxic effect against the A2058 human melanoma cell lines were observed with flavolipin [293]. In addition, serine dipeptide lipids produced by oral and intestinal Bacteroidetes bacteria are consistently recovered in lipid extracts of carotid arteries, suggesting their implication in the pathogenesis of TLR2-dependent atherosclerosis through flavolipin deposition and metabolism in artery walls [294]. Interestingly, flavolipin may be a potential biomarker of multiple sclerosis (MS), as it is expressed at significantly lower levels in the serum of MS patients compared with both healthy individuals and Alzheimer’s disease patients [295].

## 7. General conclusions

Enzymatic and nonenzymatic oxidation of PUFAs produces vast repertoires of PUFA-specific oxylipins with widespread cellular and physiological functions. In this review researchers at the forefront of their respective fields have provided overviews of the biosynthesis, structures and functions of the main classes of nonclassical oxylipins, including the recently identified FAHFAs, which are derived from saturated, monounsaturated and PUFAs. Due to the central role of PUFAs as precursors to many of these lipid mediators, recent advances in our understanding of the role of FADS in PUFA biosynthesis have also been discussed.

The overarching aim of this review is to show both the diversity of the most recently identified enzymatically and nonenzymatically-derived oxylipins, and also their roles in regulating cellular functions in health and disease. This review has provided insights into the discoveries of many new oxylipins, which have vastly extended the repertoire of fatty acid-derived bioactive lipid mediators beyond the classical eicosanoids. It has also highlighted many areas where our increased understanding of their activities may hold significant therapeutic potential. For example, in the area of SPMs, the identification of their anti-inflammatory, pro-resolving, microbial clearing, anti-thrombotic and organ-protective actions may be useful in controlling SARS-CoV-2 infection disease severity, and even long-term COVID-19 symptoms.

This review has presented a wide range of oxylipins, and shown there are potential overlapping, but also opposing actions between these diverse classes of lipid mediators, and a more integrated approach to investigating the oxylipidome and the interplay between the different oxylipins in regulating cellular functions may prove important in understanding their role in health and disease, and in the development of new therapies. An example of this was shown in the eye, where VLC-PUFAs are located at the sn-1 position and DHA the sn-2 position of retinal photoreceptor cell PC. Here, alternatively or concomitantly regulated pathways may lead to the dual formation of both ELVs and N (PD)1; however, the interplay between these oxylipins and how the different regulatory pathways are coordinated remains to be fully elucidated.

By far the greatest diversity of oxylipins is produced nonenzymatically, but as discussed above, their role and relative importance in regulating cellular functions is not well understood. There are however precedents for nonenzymatically-derived oxylipins regulating cellular responses, for example, in the activation of detoxification systems in plants [296], and ferroptosis, where phospholipid peroxidation products drive non-apoptotic cell death via an iron-dependent regulated process [297,298]. Dysregulation of ferroptosis has been implicated in a wide range of conditions, including cancer, neurodegeneration, tissue injury, inflammation, and infection [299], and as such, a greater understanding of the role of NEO-PUFAs in regulating cellular processes, such as ferroptosis, may hold great potential for the development of novel treatments.

For these reasons researchers may need to cross traditional research boundaries, and consider the wider diversity of classes of oxylipins and their inter-relationships when investigating their roles and activities to better understand the therapeutic potential of modifying the levels of specific oxylipins. To date few studies have examined the whole range of PUFA-derived oxylipins, as this analysis is complicated by a lack of commercial standards and the wide range of physiological concentrations of the different oxylipins; however, recent developments in lipidomics and mass spectrometry may make this type of analysis more feasible in the future.

There are however a number of important aspects of oxylipin metabolism that remain to be understood. For example, although the biosynthesis and activities of the different oxylipins is beginning to be characterised, there is still much to learn about the kinetics of their formation and turnover, which has been called “fluxolipidomics” [300]. There is also still much to be learned about more fundamental aspects, such as whether there are differences in the levels of the different oxylipins between males and females, and the effects of age are also not well characterised.

Further important questions relate to how responsive the different families of oxylipins are to dietary modifications. There have been very dramatic changes in both the quality and quantity of dietary fat, predominantly driven by the Agricultural and Industrial Revolutions, and culminating in the current situation where the Western diet has low levels of omega-3 PUFAs and high levels of LA, saturated fatty acids, and trans fatty acids, and has seen the omega-6 PUFA/omega-3 PUFA ratio change from around 1–2:1 to 10–20:1 [301]. As has been seen above, increased dietary LA intake can led to detrimental increases in the level of OXLAMs, and although these octadecanoids are found at higher levels in tissues and blood than oxylipins derived from any other PUFA [11], they are among the least researched and the consequences of these changes on human health need more extensive investigation. Similarly, although EPA, DPAn-3 and DHA-derived SPMs have been shown to be responsive to omega-3 PUFA supplementation, and these changes may be linked to decreases in inflammation, the exact relationship between increased intakes of EPA, DPAn-3 and DHA and increases in specific SPMs, and also ELVs, requires further research to help us progress to more precision medicine [302].

In summary, this review has provided insights into current understanding of the biosynthesis of omega-3 and omega-6 PUFAs, and the biosynthesis, structures, and functions of nonclassical oxylipins; however, further work will undoubtedly lead to the discovery of many new oxylipins, and also increase our understanding of their regulation and actions in health and disease. This review has also highlighted some of the challenges that need to be overcome in order for this research to produce clinical benefits in the diagnosis, prognosis, and treatment of diseases. These challenges include the need for a wider range of analytical standards, the lack of understanding of oxylipin kinetics and normal biological variations, and the need for greater methodological standardisation between laboratories to increase consistency in analysis of the whole range of oxylipins. These and other challenges to clinical translation have been discussed in the insightful review by Gladine and Fedorova [246]. This complex field holds significant clinical potential, and in this review we have provided an overview of some of the breadth and diversity of the different classes of oxylipins, and their importance in health and disease.

## Figures and Tables

**Table 1:** Endogenous SPMs in human tissues: mass spectrometric identification

(A) SPMs *in vivo* without intentional supplementation*			
Tissue/organ	SPMs	Reference	Country
Plasma	18-HEPE, 17-HDHA, RvE2, RvD1 17R-RvD1 and RvD2 (breast surgery)	[303]	Australia
	MaR1, RvD2, RvD4, RvD5 (adolescents) (2–4 pg/mL), 17-HDHA (~110 pg/mL), 18-HEPE (~30 pg/mL)	[304]	Switzerland, Canada
Stenotic aortic valves	RvE1, RvD3 (~500–3500 pg/g tissue)	[118]	Sweden
Sputum (cystic fibrosis)	RvD1 [~200 pg/mL (~0.5 nM)]	[305]	Italy
SARS-CoV-2 infection	RvE3, RvD1–4, PD1 (serum)	[114]	USA
	(BAL) LXA_4_, RvDs (~0.1–1.0 nM)	[113]	Canada
Knee replacement surgery	RvD2, RvE2	[306]	Australia
Gingival tissue	RvE3, RvD1, MaR1	[307]	USA
Nonobstructive coronary artery disease (WARRIOR) trial	RvD1, RvD3, RvD5, RvE1, MaR1 (~5–40 pg/mL), RvD2 ~ 1 ng/mL	[308]	USA
Chronic rhinosinusitis	RvD1, RvD2, LXA4	[309]	USA
Carotid disease (serum)	RvD1 (~80–150 pM)	[310]	USA
Bariatric surgery	RvD1 (5–8 pg/mL), RvD3 (0.6–2.4 pg/mL), RvD4 (0–240 pg/mL), PD1 (0–67 pg/mL)	[311]	USA

(B) Omega-3 PUFA supplementation increases SPMs**				
Diseases/conditions	Doses and regimens	SPMs that are increased by supplementation	Reference	Country
Chronic kidney disease (plasma)	n-3 PUFA; 4 g/day; 8 wks	RvE1, RvE2, RvE3, RvD5	[312]	Australia
In pregnancy on offspring (Plasma)	n-3 PUFA ethyl esters with DHA (56.0%) and EPA (27.7%); 3.7 g/day; from 20 wks gestation until delivery	18-HEPE, 17-HDHA	[313]	Australia
Peripheral artery disease (PAD) in OMEGA-PAD II trial (plasma)	n-3 PUFA; 325 mg EPA and 225 mg DHA per capsule; 4.4 g (4 capsules)/day; 3 months	RvE3, LXA5	[314]	USA
Major depressive disorder (plasma)	EPA 1, 2, and 4 g/d vs placebo; 12-week randomized trial	18-HEPE RvE3	[315]	USA
Postmenopausal women with chronic inflammation (plasma)	EPA and DHA; 3 g/day; two phases of 10-week supplementation, separated by a 10-week washout.	14-HDHA, 17-HDHA RvD_5n-3 DPA_ MaR1_n-3 dpa_	[316]	USA
Coronary artery disease	EPA and DHA, 3.36 g daily	RvE1, MaR1, 18-HEPE	[317]	USA
Pregnant women (Umbilical cord blood)	EPA rich fish oil (1060 mg EPA plus 274 mg DHA), or DHA rich fish oil (900 mg DHA plus 180 mg EPA)	17-HDHA, 14-HDHA	[318]	USA

* Panel A reports publications in the period of 2017–2021 that confirm and extend original findings [98].

* Panel B reports publications in the period of 2017–2021 that confirm and extend original findings [319,320].

## Abbreviations:

ARA: Arachidonic acid
COX: cyclooxygenase
CYP: cytochrome P450 mixed function oxidase
DiHODE: dihydroxyoctadecadienoic acid
DiHOME: dihydroxyoctadecamonoenoic acid
DHA: docosahexaenoic acid
DPA: docosapentaenoic acid
EPA: eicosapentaenoic acid
ELV: elovanoids
eLOX3: epidermis-type lipoxygenase 3
EDP: epoxydocosapentaenoic acid
EpETE: epoxyeicosatetraenoic acid
EET: epoxyeicosatrienoic acid
EpODEs: epoxyoctadecadienoic acid
EpOME: epoxyoctadecamonoenoic acid
FAHFA: fatty acid ester of hydroxy fatty acid
HpETE: hydroperoxyeicosatetraenoic acid
HDoHE: hydroxydocosahexaenoic acid
HEPE: hydroxyeicosapentaenoic acid
HETE: hydroxyeicosatetraenoic acid
HODE: hydroxyoctadecadienoic acid
HOTrEs: hydroxyoctadecatrienoic acid
LOX: lipoxygenase
MaR: maresin
NEO-PUFA: non-enzymatically oxidized PUFA
(N)PD: (neuro)protectin
OXLAM: oxidized linoleic acid metabolite
OGD: oxygen-glucose deprivation
PUFA: polyunsaturated fatty acid
Rv: resolvin
SASP: senescence-associated secretory phenotype
sEH: soluble epoxide hydrolase enzymes
SPM: specialized pro-resolving mediator
TAG-Est: triacylglycerol-estolides.
